# The landscape of the long non-coding RNAs in developing mouse retinas

**DOI:** 10.1186/s12864-023-09354-w

**Published:** 2023-05-10

**Authors:** Dongliang Yu, Yuqing Wu, Leilei Zhu, Yuying Wang, Donglai Sheng, Xiaofeng Zhao, Guoqing Liang, Lin Gan

**Affiliations:** 1 College of Life Sciences and Medicine, Zhejiang Sci-Tech University, Hangzhou, Zhejiang 310018 China; 2 Institute of Life Sciences, Hangzhou Normal University, Hangzhou, Zhejiang 310036 China; 3 Department of Neuroscience & Regenerative Medicine, Medical College of Georgia, Augusta University, Augusta, GA 30912 USA

**Keywords:** lncRNA, Retina, Spatiotemporal specificity, Co-expression, Noncoding regulator

## Abstract

**Background:**

The long non-coding RNAs (lncRNAs) are critical regulators of diverse biological processes. Nevertheless, a global view of its expression and function in the mouse retina, a crucial model for neurogenesis study, still needs to be made available.

**Results:**

Herein, by integrating the established gene models and the result from ab initio prediction using short- and long-read sequencing, we characterized 4,523 lncRNA genes (MRLGs) in developing mouse retinas (from the embryonic day of 12.5 to the neonatal day of P28), which was so far the most comprehensive collection of retinal lncRNAs. Next, derived from transcriptomics analyses of different tissues and developing retinas, we found that the MRLGs were highly spatiotemporal specific in expression and played essential roles in regulating the genesis and function of mouse retinas. In addition, we investigated the expression of MRLGs in some mouse mutants and revealed that 97 intergenic MRLGs might be involved in regulating differentiation and development of retinal neurons through Math5, Isl1, Brn3b, NRL, Onecut1, or Onecut2 mediated pathways.

**Conclusions:**

In summary, this work significantly enhanced our knowledge of lncRNA genes in mouse retina development and provided valuable clues for future exploration of their biological roles.

**Supplementary Information:**

The online version contains supplementary material available at 10.1186/s12864-023-09354-w.

## Introduction

Long non-coding RNAs (lncRNAs) are critical regulatory elements in eukaryotic organismsthat could modulate gene expression during diverse developmental, physiological, and pathological processes [1, 2]. With the wide application of high-throughput sequencing and the development of computational tools [3], a rapidly growing number of lncRNAs have been identified in the last decade [4, 5]. However, due to the high specificity of lncRNAs in spatiotemporal expression, continuous effort is still required to comprehensively understand lncRNA expression and biological functions in particular tissues and developmental stages [6].

The vertebrate retina is an important model system for neurogenesis study. It mainly contains six principal types of neuron cells generated from retinal progenitor cells (RPCs) under complex and precise regulation [7]. In addition to the transcription factors like homeobox proteins (e.g., Pax6, Isl1, and Vsx2), bHLH proteins (e.g., Neurod1, Mash1, and Bhlhe23), and POU-domain proteins (e.g., Pou4F1 and Pou4F2) [8, 9], the critical regulatory role of lncRNAs in fate determination and development of retinal neuron cells and visual maintenance are also documented by increasing evidence. For instance, *Six3os1* and *MIAT* (also known as *Rncr2*) regulate the specification or differentiation of photoreceptors, bipolar cell, amacrine cell, and Müller glial in mouse retina [10, 11]. While lncRNAs *ZNF503-AS1*, *MALAT1* and *lnc-SCA7* are associated with retinal diseases such as age-related macular degeneration, glaucoma, and diabetic retinopathy, through the ways like regulating the differentiation or degeneration of retinal pigment epithelial and neurons [12–14]. For more information, please refer to the excellent reviews by Wan et al. and Sun et al. [15, 16].

Until recently, some genome-wide studies have been carried out to investigate the lncRNA expression in the mouse retina. For example, Chen et al. demonstrated the tissue and stage specificity of lncRNA expression in six ocular tissues through microarray analyses and postulated the possible function of prominently expressed lncRNAs in regulating neural development [17]. During the preparation of this manuscript, we also noticed that through whole transcriptome sequencing and analyses, Chen et al. revealed the expression of about 2,600 lncRNAs in the developing mouse retinas (E14.5, P1, P7, P12, P17, and adult mice) and proposed critical roles of the lncRNAs *Meg3* and *Vax2OS* in regulating retinal development through circRNA/lncRNA-miRNA-mRNA network [18]. However, these works mainly focused on those well-characterized genes, and there needs to be more effort toward a *de novo* annotation of retinal lncRNAs. In 2019, based on the full-length transcripts derived from iso-seq of mixed mouse retinas (E12.5 to P28), we identified 940 intergenic lncRNAs (lincRNAs), with most of them were novel isoforms or transcribed from novel loci [19]. Nevertheless, as have been stated then, limitations raised by its sequencing strategy, e.g., mixed RNAs were used and relatively low coverage of reads were generated, make it still difficult to estimate the overall genomic features of retinal lncRNAs and their dynamics expression through the retina development.

In the present work, through combined analyses of the data from short-read lncRNA sequencing, long-read isoform sequencing, and established gene models from genome annotation, we generated the most comprehensive collection of mouse retinal lncRNAs. In addition, we assessed the expression of these lncRNAs in different developmental stages and tissues and in several mutants that are linked to abnormal development of retinal neurons, which significantly promoted our knowledge of the retinal lncRNAs’ genomic features and molecular functions.

## Results

### Identification of the mouse retinal lncRNAs

**Fig. 1:**
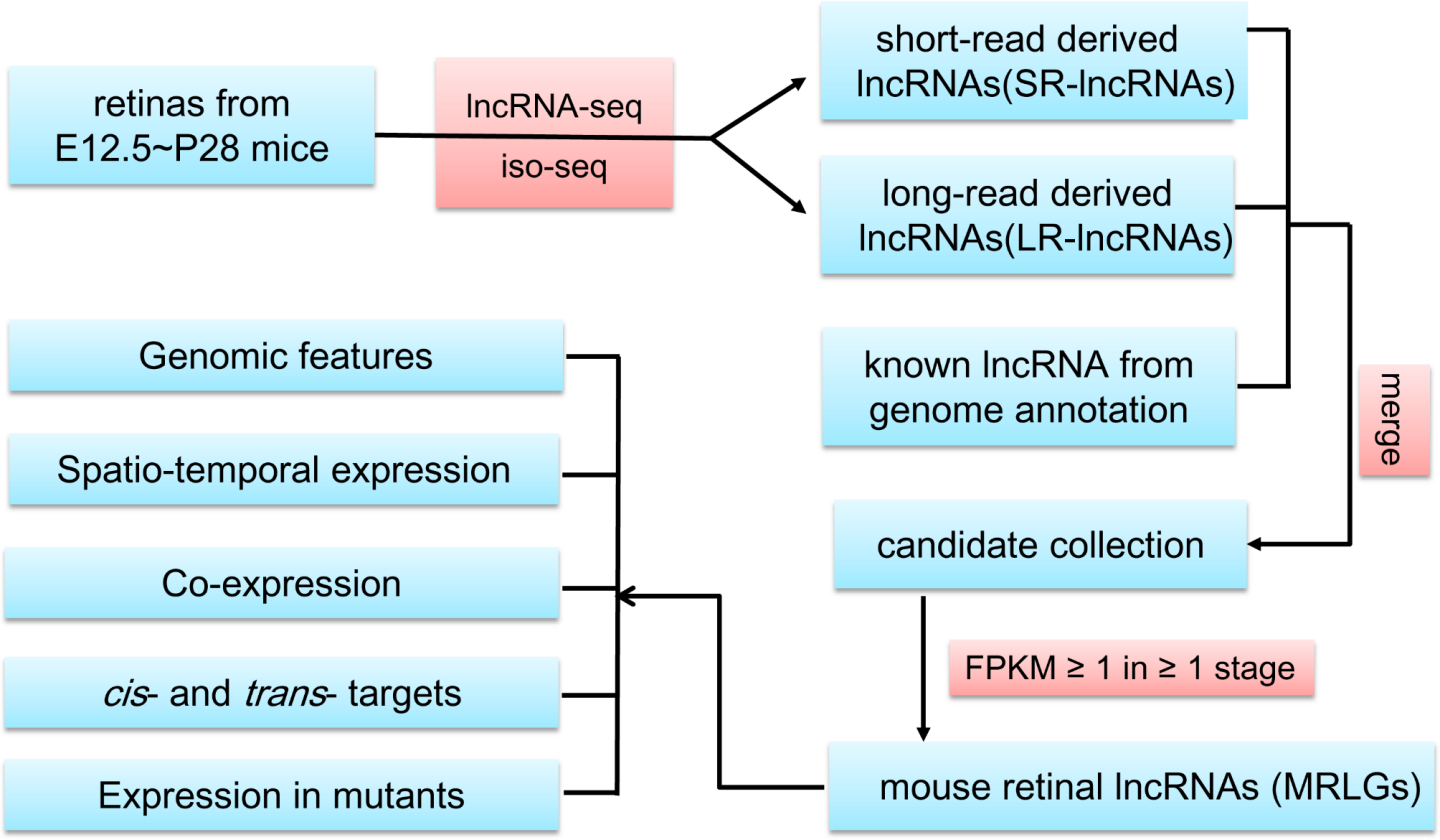
The workflow of lncRNA annotation and further analyses. This work merged the lncRNA transcripts from lncRNA-seq, iso-seq, and genome annotation to generate a systematic annotation of lncRNA genes expressed in developing mouse retinas

In this work, we performed lncRNA identification using a hybrid strategy (Fig. 1). First, strand-specific libraries were constructed and sequenced for mouse retinas from fifteen developmental stages starting from embryonic day 12.5 (E12.5) to postnatal day 28 (P28), respectively. The alignment of these short reads to the reference genome and the subsequent transcriptome reconstruction gave rise to 326,239 transcripts transcribed from 260,539 gene loci. After filtering out the single-exon encoded transcripts and transcripts that overlapped the exons of annotated protein-coding genes or showed detectable coding potential, we acquired 2,514 multiple-exon encoded noncoding transcripts from 1,925 loci (hereafter referred to as Short Reads derived lncRNAs, SR-lncRNAs).

In addition, we re-analyzed the full-length transcripts generated from iso-seq of the cDNA library of mixed retina tissues to identify the lncRNA candidates. The transcripts of known coding genes or detected coding potential were filtered out, resulting in 2,981 multiple-exon encoded noncoding transcripts from about 2,495 loci (hereafter referred to as Long Reads derived lncRNAs, LR-lncRNAs).

To generate an annotation of retinal lncRNA genes as much intact as possible, we merged the SR- and LR-lncRNAs and the lncRNAs from the genome annotation (VM22-lncRNAs), which generated 22,695 lncRNAs transcribed from 16,298 gene loci. After that, we removed the lowly expressed genes (FPKM < 1) in all the investigated stages. Finally, we acquired a collection of 4,523 lncRNA genes that were considered the “Mouse Retinal LncRNA Genes” (MRLGs) for further analyses (Table S1).

**Fig. 2:**
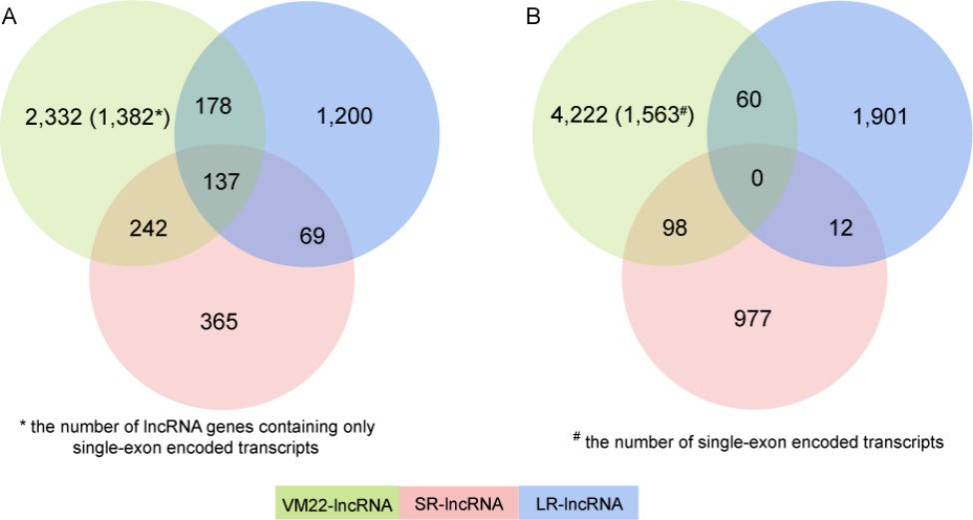
The contribution of different gene sets to mouse retinal lncRNA identification. The lncRNA genes (A) and transcripts (B) derived from lncRNA-seq, iso-seq, and genome annotation were compared to the final collection of mouse retinal lncRNAs. The genes were counted as overlapping if they were encoded by overlapping genomic regions and transcribed from the same strand. In contrast, the transcripts were counted as overlapping when they showed the same exon-intron structures

The contribution of three independent data sources, i.e., SR-lncRNAs, LR-lncRNAs, and VM22-lncRNAs, to the final MRLG annotation was subsequently assessed (Fig. 2). From gene identification, we found that VM22-lncRNAs contributed the largest number of MRLGs (2,889), including 2,332 specific gene loci. Mainly, this was ascribed to single-exon encoded lncRNA transcripts (SE-lncRNAs) being exclusively collected from VM22-lncRNAs in our strategy, i.e., 1,382 out of these 2,332 loci only contained SE-lncRNAs. In contrast, SR-lncRNAs and LR-lncRNAs identified 2,191 lncRNA gene loci, including 1,634 not reported in the genome annotation. Furthermore, long reads contributed more than short reads in gene identification, i.e., 1,200 MRLGs were specific to LR-lncRNAs, while only 365 to SR-lncRNAs (the transcripts of the lncRNA genes were only detected from the reconstructed transcriptomes derived from long reads or short reads). Similarly, for the 7,300 transcripts transcribed from the MRLG loci, most were descended from genome annotation, while 1,973 and 1,087 were evidenced by the full-length transcripts derived from iso-seq and the reconstructed transcripts derived from lncRNA-seq, respectively.

Notably, MRLG annotation found many novel isoforms for known lncRNA genes. For 430 VM22-lncRNA corresponding loci in the final MRLG annotation, iso-seq and lncRNA-seq respectively found 408 and 433 novel isoforms. Taking the genes that were most significantly varied in isoform numbers for example, we detected nearly all known isoforms (10/11) and 21 novel isoforms of the gene *Six3os1* (*XLOC_006735*), as well as four out of ten known isoforms and 11 novel isoforms of *Firre* (*XLOC_015623*).

### Genomic and expression features of the MRLGs

**Fig. 3:**
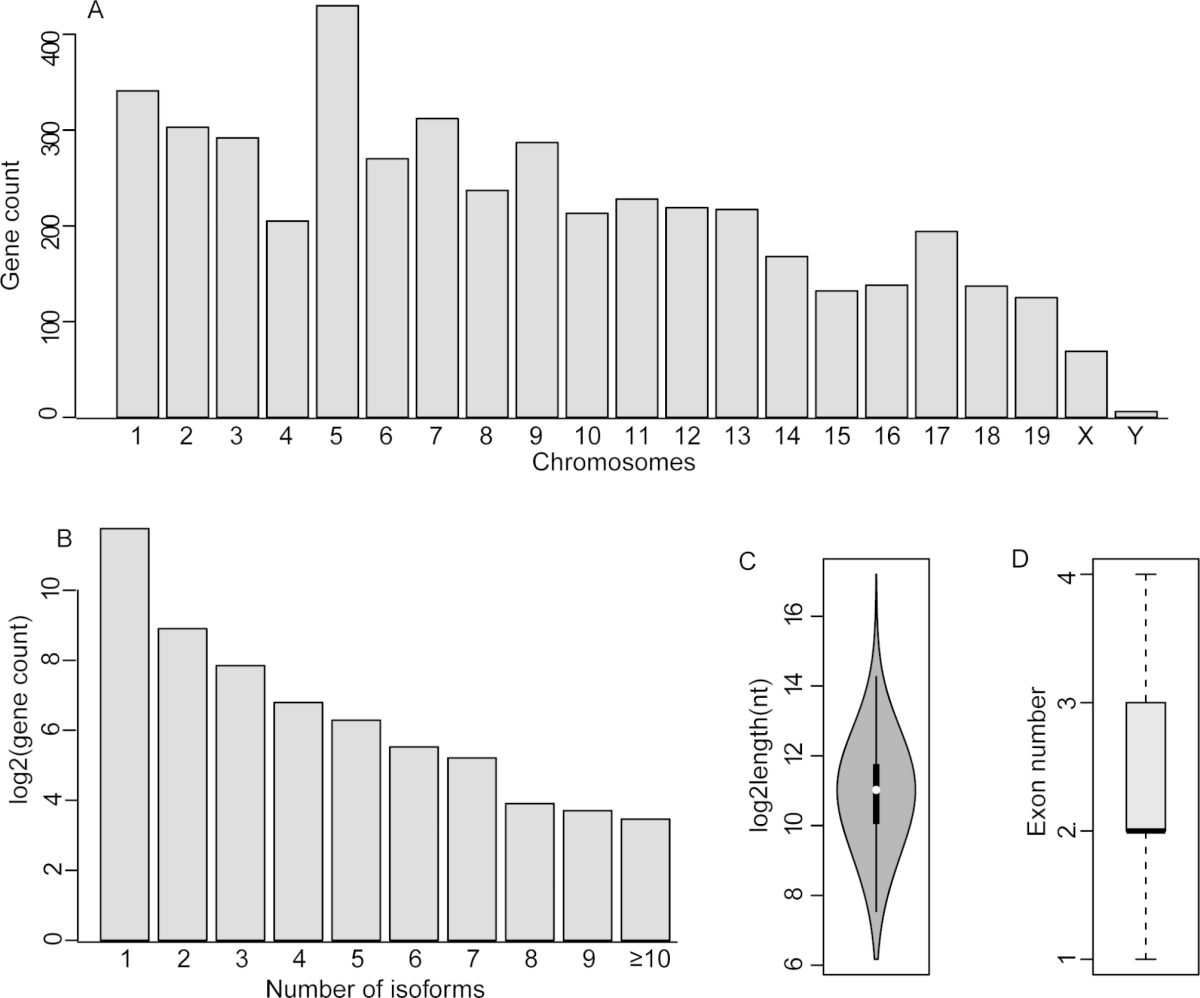
The genomic features of mouse retinal lncRNAs. (A) Distribution of the lncRNA genes in chromosomes. (B) The number of total identified isoforms of expressed lncRNA genes (not restricted to the isoforms identified in mouse retinas). (C) and (D) Statistics of the length and exon number of lncRNA transcripts

The MRLGs were distributed scattering through the whole genome, with chr5 (430) and chrY (6) containing the largest and smallest numbers, respectively (Fig. 3A). Even if isoforms collected from the genome annotation, which might not be expressed in the retina, were included, fewer than three isoforms were found for most MRLGs (Fig. 3B). In addition, the average length of the MRLG transcripts (ca. 2,900 nt) was much longer than that of the previously known mouse lncRNAs (ca. 1,380 nt), which probably benefited from the integration of long-read sequencing data in this work (Fig. 3C). 1,563 MRLGs only contained SE-transcripts, while the others contained at least one ME-transcripts with an average exon number of 3.2 (ranging from 2 to 33) (Fig. 3D). According to the relative position to protein-coding genes, MRLGs were classified into five groups, i.e., antisense (1,319), divergent (330), convergent (229), intronic (2,089), and strict lincRNAs (556).

Based on the 60-way multiple species alignment of vertebrate genomes, we found that the conservation of MRLGs exons was lower than coding exons but slightly higher than the random genomic regions (Figure S1). In addition, a primary sequence-based homology search revealed that 1,423 MRLGs (31.5%) were conserved in evolution, including 385 in more than two vertebrates, such as the well-known genes *Miat*, *Malat1*, *Tug1*, and *Meg3*. Furthermore, rat and human share 1,093 and 402 MRLGs with the mouse, respectively, in contrast to less than 100 in several other species, such as zebrafish, chicken, and platypus (Figure S2).

**Fig. 4:**
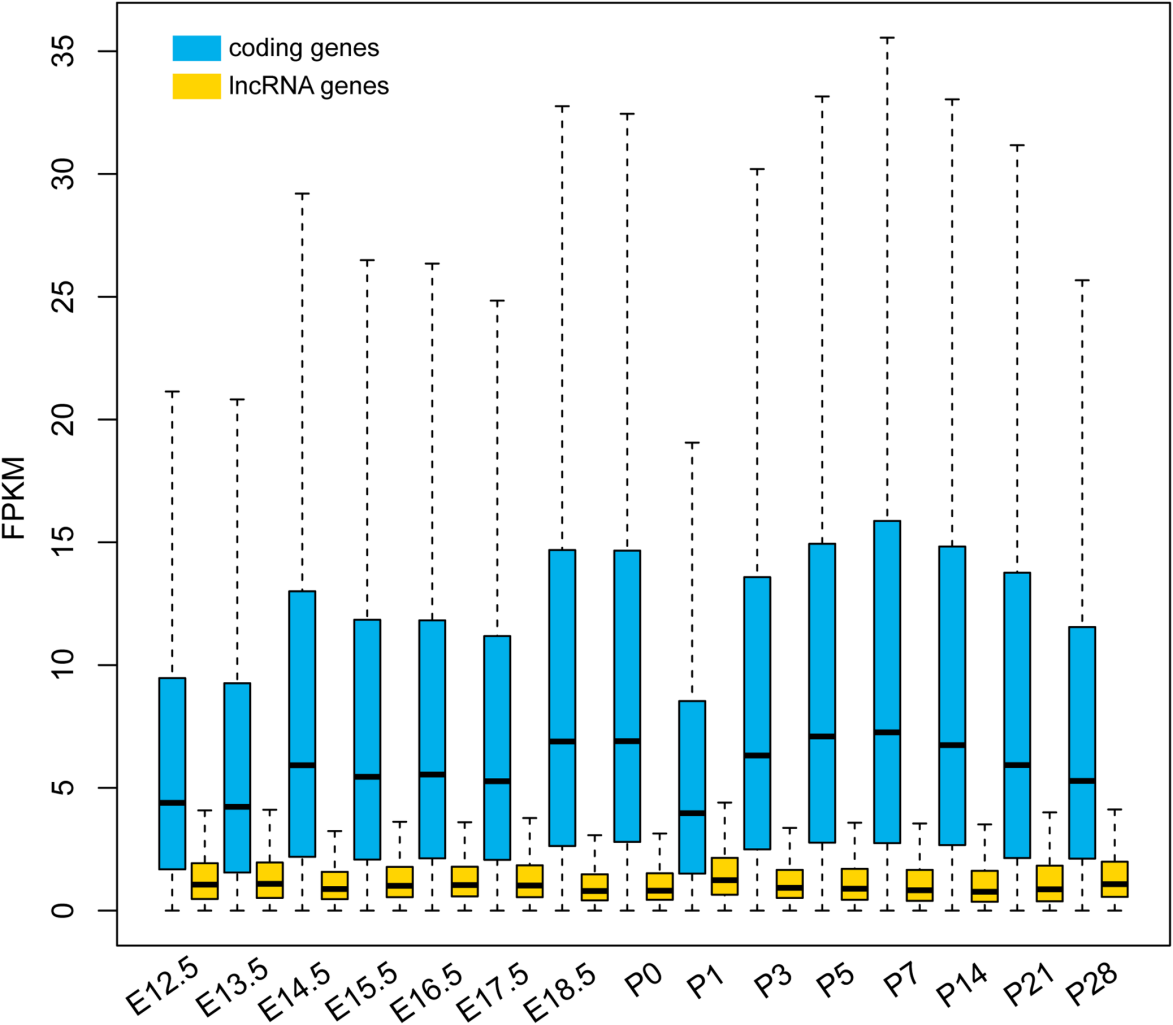
Expression levels of protein-coding genes and lncRNA genes in developing mouse retinas. E: embryonic; P: postnatal

Derived from the lncRNA-seq data analyses, we obtained an FPKM matrix consisting of the expression levels of the protein-coding genes and MRLGs in developing mouse retinas. It appeared that MRLGs were expressed at a lower level than coding genes (Fig. 4), with their median FPKM values ranging from 0.8 to 1.2 and 4.0-7.3 in the investigated samples, respectively. Notably, the content of the highest expressed MRLGs in individual samples was much the same, e.g., the expression of *Rmrp*, *Gm28960*, *Gm37750*, and *Gm43940* were the top ten in all stages. In addition, the MRLGs showed higher stage specificity than the protein-coding genes (Figure S3) (*t*-test, *p*-value < 0.001). For example, 2.1% of the MRLGs (94/4523) appeared to be stage-specific (τ ≥ 0.8), which was significantly more so than protein-coding genes (1.3%, 168/13,187).

We further compared the expression of MRLGs of different classes and found that the intronic MRLGs were most varied from the others (Figure S4). In contrast, expression levels between convergent and other MRLGs (i.e., antisense, divergent and intergenic lncRNAs), as well as between divergent and intergenic MRLGs, were very similar in most stages. Interestingly, expression variation between classes was rarely observed in some stages, such as E18.5, P0, P3, and P28.

### Identification and function analyses of the cis- and trans-targets

Regulatory roles of lncRNAs could perform through *cis-* and *trans-*acting mechanisms. The *cis-*targets of lncRNAs usually refer to the nearest coding genes in the same chromosome. In contrast, the *trans*-regulation is not dependent on the relative position of lncRNA and targets in chromosomes. In this work, we identified 894 nearest protein-coding genes within 100 kb genomic regions up or downstream of the MRLGs. To increase the specificity of *cis*-targets identification, only 74 protein-coding genes that showed strong expression correlation (*cor* ≥ 0.9 and *p*-value ≤ 0.05) to their nearby lncRNAs were herein classified as the *cis*-targets. Functional analyses revealed the ‘retinal ganglion cell axon guidance’ (GO:0031290) related genes were enriched in the *cis*-targets (χ^2^-test, *p*-value ≤ 0.05), including *Pou4f2* (*Brn3b*), *Efna5* and *Zic2*. Besides, the *cis*-targets also enriched genes related to cell differentiation and anatomical structure development, such as the homeobox transcription factors *Tgif2*, *Lmx1b* and *Meis2* (Table S2).

On the other hand, we characterized the *trans*-targets of MRLGs through co-expression analyses. The protein-coding gene was determined as a *trans*-target of one MRLG if they were strongly correlated in expression and were clustered into the same WGCNA module. As a result, 133,371 *trans*-regulations between 1,223 MRLGs and 6,976 protein-coding genes were determined. Subsequently, the biological roles of 796 MRLGs were predicted based on the *trans*-targets. It revealed that more than 10% of them were involved in biological processes such as vesicle-mediated transport in the synapse, visual perception, autophagy, and detection of light stimulus, indicating their close association with the physiological roles of retina tissue (Figure S5).

### Temporary expression of MRLGs in developing mouse retinas

**Fig. 5:**
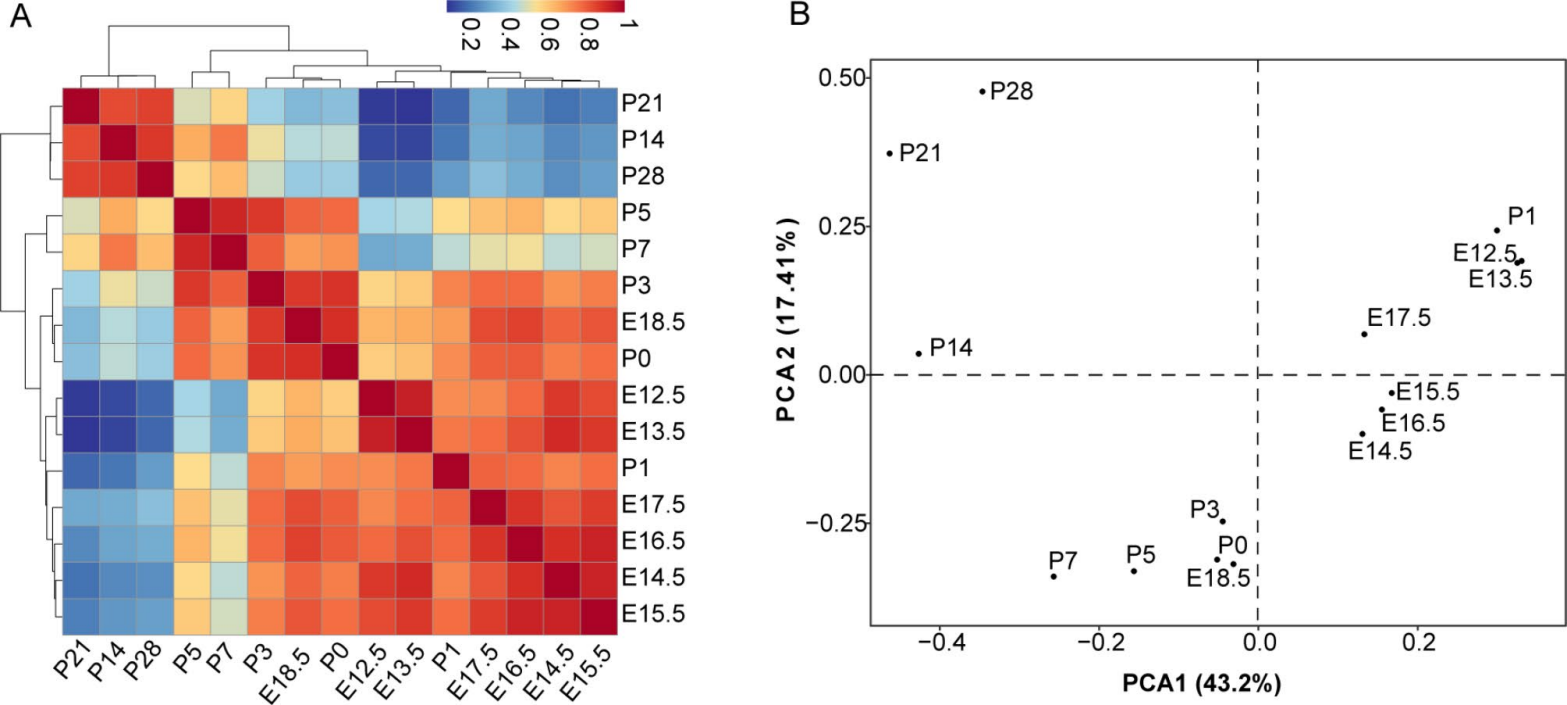
Hierarchical clustering and PCA analyses of developing mouse retinas. (A) Clustering analyses of developing retinas was based on the pairwise Spearman’s correlation coefficient of lncRNA gene expression. (B) The PCA analyses of the mouse retinas suggested that the lncRNA expression was significantly modulated around the birth event

Based on the expression profiles, pairwise Pearson correlation coefficients between stages were calculated, which clustered the developmental stages into two main groups. According to the majority, they were hereafter referred to as the embryonic group (E12.5-E17.5) and neonatal group (E18.5-P28) (Fig. 5A). Generally, the MRLG expression profiles in adjacent periods showed a higher correlation. Interestingly, the P1 profile was more similar to the early days of retinal neurogenesis (E12.5 and E13.5) but not the adjacent period P0 or P3. Additional principal component analyses (PCA) also revealed this pattern according to PCA1 (Fig. 5B).

To assess the variation of lncRNA content in developing retinas, we categorized the MRLGs into ‘expressed’ or ‘not expressed’ in individual samples with an empirically strict cutoff (FPKM ≥ 1 in more than one stage). As a result, about 15% MRLGs were detected in all the stages, while a similar number of MRLGs (704) were specifically expressed (Figure S6). For individual stages, the number of expressed MRLGs varied greatly, i.e., from about 1,800 in E18.5, P0, and P14 to 2,774 in P1. Meanwhile, for any two stages, the number of shared lncRNAs ranged from 41% (between P1 and P14) to 95% (between E14.5 and E15.5) (Table S3). The E12.5 and E13.5 retinas shared the most MRLGs (2,169 genes), accounting for about 90% of their lncRNA contents. In contrast, the E12.5 and P14 retinas shared the fewest MRLGs (1,004), with only about 42% and 55% of the lncRNAs expressed in these two stages, respectively.

**Fig. 6:**
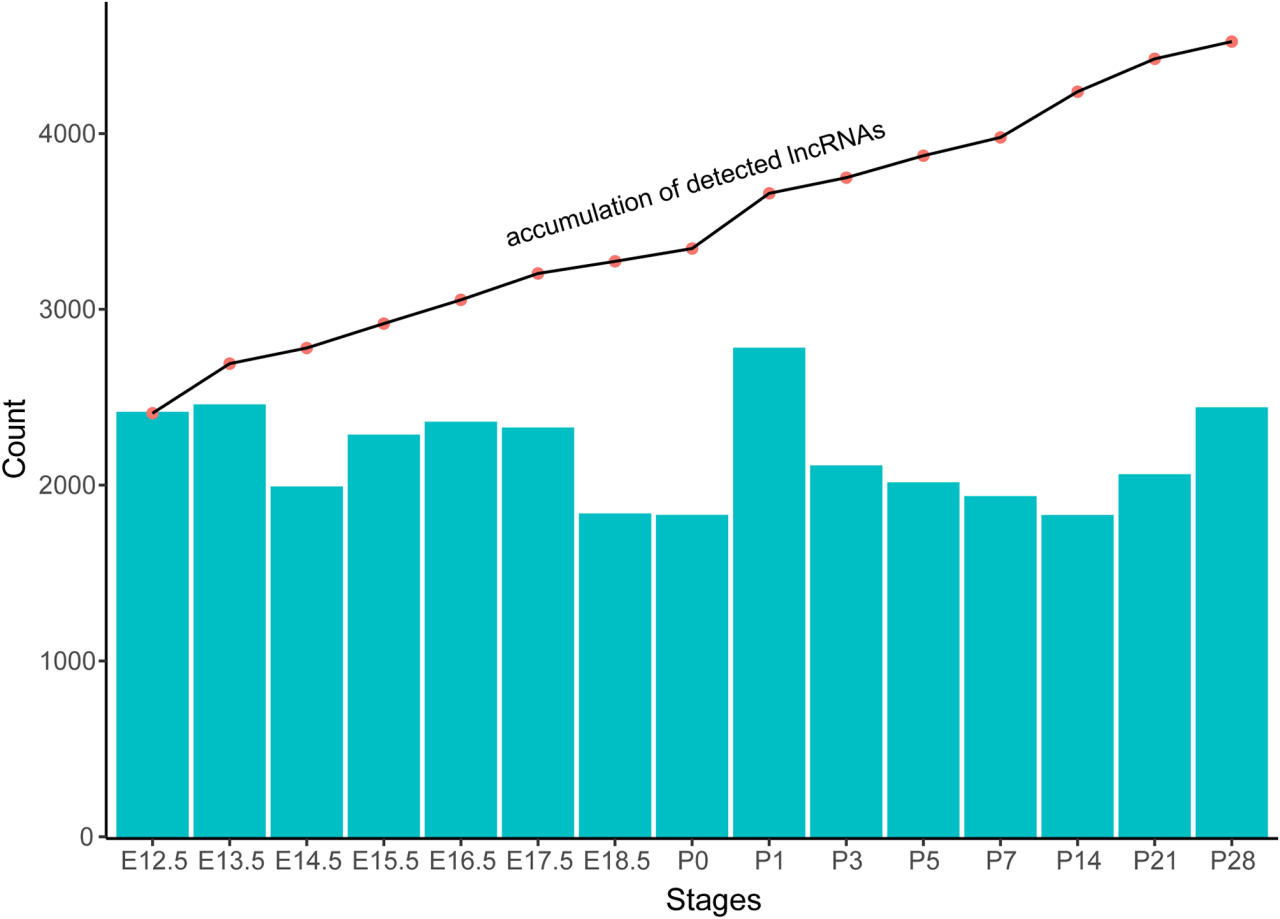
Accumulation of detectable lncRNA genes along with the mouse retina development. The bar chart indicates the number of lncRNA genes expressed at each stage and the line chart shows the accumulated number of detected lncRNA genes from the day of E12.5

Furthermore, we found that about 100 additional MRLGs were detected in each stage after E12.5 according to the chronological order of development (Fig. 6). Exceptionally, 282 and 314 additional MRLGs were identified at E13.5 and P1, respectively. Interestingly, the detected MRLGs were much more in P1 (2,774) than in P0 (1,823) retinas, but only 314 additional lncRNA genes were identified from the period P0 to P1. An inspection of the shared MRLG contents between P1 retinas and others showed that E13.5 and E16.5 retinas shared the largest number of lncRNAs with P1. Still, in E17.5 and E18.5 retinas, the shared MRLG contents were largely decreased, suggesting that many MRLGs were down-regulated at or before P0 (*e.g.*, E17.5 or E18.5) and up-regulated at P1.

### Modulation of MRLGs’ roles during retina development

As mentioned above, MRLG content was altered a lot during retina development. However, due to the function ambiguity of most MRLGs, their association with the modulation of tissue physiological features during development remains unclear. In this work, through the co-expression analyses, we found about 560 to 880 *trans*-acting lncRNAs were expressed in individual retina samples, representing about 26–39% of the total MRLGs. Thus, herein we tried to describe the general roles of MRLGs in developing retinas through these *trans*-acting lncRNAs.

First, functional enrichment analyses of the targets (coding genes) were performed for each *trans*-acting lncRNA having three or more targets. Then, the functions of these lncRNAs were assigned by the top five enriched GO terms. We subsequently summarized the presented GO terms and their abundance in a matrix. Further clustering analyses based on the matrix revealed a close association between developmental stage and lncRNA function. It appeared that the main roles of lncRNAs continuously altered along with retina development. Consistent with gene expression profiles, more similar functions were also observed for lncRNAs expressed in adjacent periods. In contrast, significant difference was observed there between the early embryonic (E12.5/E13.5) and neonatal retinas (P14-P28) (Figure S7).

To further describe the variation of lncRNAs’ roles during retina development, we ranked the GO terms in each stage by their abundance (Table S4). It showed that the MRLGs involved in ‘Golgi vesicle transport’ (GO:0048193) and autophagy related biological processes (GO:0006914 and GO:0061919) were abundant in both embryonic and neonatal retinas, although dropped in the rankings from P5. In contrast, the rankings of some other GO terms rise along with the retina development, e.g., ‘sensory perception of light stimulus’ (GO:0050953) moved up from 24th at E12.5 to 1st after P5, as well as ‘synaptic vesicle cycle’ (GO:0099504) and ‘vesicle-mediated transport in synapse’ (GO:0099003) were about 30th at E12.5 and came in top ten around the day of birth. On the contrary, the processes associated with microtubule-based movement and transport were significant in the early embryonic days, but their proportions were strikingly decreased around P3-P5. These facts revealed that the dynamic expression of MRLGs was associated with the process of establishing the functional retinas and the MRLGs might be involved in various processes, including neuron cell differentiation, development, migration and function.

### Tissue specificity of MRLG expression

Expression of lncRNAs usually shows extensive tissue specificity, which is also significantly associated with the tissues’ physiological roles. Herein, we analyzed the MRLG expression in 15 mouse tissues and found that 2,004 MRLGs were expressed in at least one of these tissues, including 73 expressed in all (e.g., the well-known lncRNAs *Gas5*, *Tug1*, and *Malat1*). The nervous tissue cerebrum expressed the largest number of MRLGs (1,003), followed by the thymus (797), testis (764) and mammary gland (744). In contrast, the digestive tissues, such as the stomach, liver and intestines, expressed the fewest number of MRLGs (< 400) (Figure S8).

498 MRLGs were only expressed in the retina and cerebrum, which were enriched in biological processes such as excitatory synapse assembly and its regulation (GO:1,904,861 and GO:1,904,889), postsynaptic density organization (GO:0097106), and eye morphogenesis (GO:0048592) (χ^2^-test, *p*-value ≤ 0.05). Likewise, according to our predicted cis-regulations, six of these MRLGs possibly *cis*-regulated their adjacent coding genes, i.e., *XLOC_000612*-*Rgs20*, *XLOC_001325*-*Marcks*, *XLOC_006138*-*Olig2*, *XLOC_006289*-*Glo1*, *XLOC_007869*-*Lrrc55* and *XLOC_015511*-*Zxdb*. Notably, the involvement of the *cis*-targets Marcks and Olig2 in retina development has been previously reported. For example, disruption of Marcks would result in defects in retinal lamination in mice [20], and Olig2 is one of the markers for retinal progenitor cells and some differentiated retinal subpopulations [21].

In addition, 2,519 MRLGs (55.7%) were lowly expressed in tissues other than the retina and were considered retina-specific. Comparative analyses revealed that they were expressed at a lower level than those non-specific ones on the whole (*t*-test, *p*-value: 5.6E-17). When checking their expression in individual stages, we found this variation was particularly evident from the day of E18.5 (Figure S9). Furthermore, from the aspect of functions, retina-specific MRLGs were over-represented in the biological process of ‘negative regulation of fibroblast cell migration’ (GO:0010764) (χ^2^-test, *p*-value ≤ 0.05).

### Differential expression of MRLGs induced by function loss of some transcription factors

To further understand the biological roles of the MRLGs, we analyzed their expression in the retina tissues of several gene knockout mutants. Due to the collected RNA-seq data are not derived from sequencing of strand-specific libraries, only the strict lincRNAs were investigated here to minimize the errors arising from the overlap of lncRNAs and mRNAs. As a result, we identified 97 intergenic MRLGs that were differentially expressed after function loss of Math5, Isl1, Brn3b, NRL, Onecut1 (Oc1) and Onecut2 (Oc2) (Table S5).

We found two and three intergenic MRLGs were varied in expression in Brn3b and Isl1 knockout mutants (E14.5), respectively, in contrast to 18 in the Math5-null mutant (E14.5). Notably, *XLOC_013893* (*Gm28511*) was down-regulated after the function loss of all three TFs. According to the dynamic expression profiles, expression of *XLOC_013893* peaked around E14.5 and was undetectable in the retinas after birth. Moreover, we found that *XLOC_013893* is about 64 kb away from its potential *cis*-target *Irx3*, which two showed strong expression correlation during retina development (*cor* = 0.74, *p*-value: 3.67E-06). The *Irx3* gene was also significantly down-regulated in the retinas of Math5-, Isl1-, and Brn3b-null mutants (log2foldchange about − 3.0 to -2.3). Comparative genomics analyses showed that *XLOC_013893* is conserved in the human genome and that the full-length transcript sequence could be mapped to chr16 with an identity of 93.5%. The corresponding genomic region in the human genome encoded an RNA gene *ENSG00000287885*, which is also close to the *Irx3* gene (about 83 Kb away), suggesting the *XLOC_013893*-*Irx3* regulation is conserved between human and mouse.

Oc1 and Oc2 are involved in the fate determination of early retinal cell types, such as horizontal cells (HCs), RGCs, cones and amacrine cells [22]. Seven and one intergenic MRLGs were differentially expressed in E14.5 retinas after function loss of Oc1 and Oc2, respectively, while six were found after double knockout of Oc1 and Oc2 (Oc1/Oc2 DKO). Interestingly, rare DEGs were shared by Oc1-null, Oc2-null and DKO mutants. The biological function of these differentially expressed lncRNAs was not determined. However, while checking their potential trans-targets, we found that two were possibly associated with the visual perception (*XLOC_010064*, *p*-value: 1.14E-19) and vesicle-mediated transport in the synapse (*XLOC_011622*, *p*-value: 3.04E-08).

In addition, we assessed the expression of intergenic MRLGs in adult NRL-null mutants (P21). The neural retina leucine zipper protein NRL is required for rod photoreceptor development. Its function loss resulted in down- and up-regulation of 44 and 25 intergenic MRLGs, respectively. Inferred from the functions of *trans*-lncRNAs, we found that these 69 differentially expressed MRLGs were closely associated with muscle system process (GO:0003012), regulation of membrane potential (GO:0042391), and sensory perception of light stimulus (GO:0050953). We also found three *cis*-targets for DEGs, i.e., *XLOC_007186-8030462N17Rik*, *XLOC_011105-Lcorl*, and *XLOC_012109-Strip2*, but the involvement of these lncRNAs and their *cis*-targets in retina development remains still unclear.

Some transcription factors are redundant in biological functions, such as Brn3b and Isl1 in RGC differentiation and survival, as well as Oc1 and Oc2 in the fate determination of several early retinal cell types. However, few intergenic MRLGs were regulated in the same manner in their function loss mutants, i.e., we only found that *XLOC_013893* were down-regulated in both Brn3b- and Isl1-null mutants, *XLOC_005930* were up-regulated in Oc1- and Oc2-null mutants, as well as *XLOC_002920*, was up-regulated in Oc1-null and Oc1/Oc2 DKO mutants. Notably, *XLOC_010659* was down-regulated in both Math5- and Oc1-null mutants, which might be consistent with the role of Math5 and Oc1 in regulating the differentiation of early cell types. Likewise, we also found some lncRNAs were differentially regulated among mutants, e.g., *XLOC_011005* was down-regulated in Oc1- and Math5-null mutants but was up-regulated in NRL-null mice, *XLOC_011568* was up-regulated in Oc1-null mutant but down-regulated in Math5-null mice, and *XLOC_011622* was up-regulated in Oc1/Oc2 DKO but down-regulated in NRL-null mutants.

## Discussion

### Utilization of time-series samples and combined sequencing technologies drive a more comprehensive annotation of mouse retinal lncRNAs

With the deepening understanding of the biological roles of lncRNA genes in eukaryotic cells, increasing efforts have been made to its identification, expression features, and interaction mechanisms with other RNAs or proteins. Analyzing the reconstructed transcriptomes derived from high-throughput sequencing is currently the most commonly used strategy for genome-wide identification of the lncRNAs. Due to the substantial progress of sequencing technology, i.e., a much lower cost for the generation of massive data and the application of long-read sequencing, more sequencing projects are now carried out, and a growing number of lncRNAs are identified in a broader range of organisms. Notably, the records of lncRNA genes continuously and largely increase when more data sources are available, even in the model organisms like the mouse, suggesting it is still a challenge for a full view of lncRNA expression in eukaryotic cells.

In this work, we identified 4,523 lncRNA genes (MRLGs) in developing mouse retinas, which showed high spatiotemporal specificity in expression. For example, more than half MRLGs were potentially retina-specific and were not detected in the other fifteen tissues. Meanwhile, we found that only about 15% MRLGs were expressed in all the stages from E12.5 to P28. Between the most deviate stages P1 and P14, up to 60% of the MRLGs were stage-specific. In addition, according to the timeline, about 3–5% more MRLGs were identified when additional samples were available. These facts indicated that using multiple and time-series samples in this work is undoubtedly helpful and necessary to minimize the adverse effect of high expression specificity on lncRNA identification.

Except for the sample size, the hybrid utilization of short- and long-read sequencing platforms also largely improved the lncRNA identification. Strand-specific RNA-seq has been widely used in lncRNA identification for its advantage in determining the transcribed strand and distinguishing the antisense transcripts from the overlapping genes [23, 24]. However, in contrast to long-read sequencing that directly gives rise to many full-length transcripts, short reads generated from the RNA-seq usually represent transcript fragments. Thus, assembling short reads into transcripts is always required before further use of the RNA-seq data, and the result of RNA-seq based lncRNA annotation is greatly limited by the accuracy of transcriptome reconstruction. In this work, we identified about 2,000 SR-lncRNA and 2,500 LR-lncRNA genes, but an extraordinarily small number of them overlapped. Furthermore, the transcripts of LR-lncRNA genes are much longer than so of SR-lncRNA genes, and the long reads covered MRLGs were commonly expressed at a higher level than those only covered by short reads. These facts suggested a better performance of RNA-seq in identifying the relatively lower expressed genes, but the long-read sequencing is well performed in gene identification. Accordingly, a more widespread application of long-read sequencing in identifying lncRNAs is indispensable for enhancing our understanding of their transcriptional complexity.

### High expression specificity of MRLGs indicated their crucial regulatory roles in retina development

High spatiotemporal specificity is one of the main features of lncRNA expression and has been widely observed in eukaryotic organisms. Taking the mouse retina for example, Chen et al. (2017) analyzed the lncRNA expression in P0 and 8-week-old mouse eyes using microarray, which showed 910 and 686 lncRNAs were specifically expressed in the ocular tissue subsets including cornea, lens, retina, RPE, choroid, and sclera. In addition, our previous work indicated the RGC-specific expression of three lincRNAs (*linc-3a*, *-3b*, and *− 3c*) through fluorescence in situ hybridization (FISH). More recently, Ayupe et al. investigated the lncRNA expression in injury-resilient (ipRGCs) and injury-susceptible RGCs (ooDSGCs) and revealed the subtype-specific expression of a large proportion of lncRNAs, i.e., 31% (268/851) and 49% (564/1147) in ipRGCs and ooDSGCs, respectively [25]. Together these demonstrated high specificity of lncRNA expression at the levels of tissue subset, as well as neuron type and subtype, and suggested the critical regulatory roles of lncRNAs in retina development.

Based on the annotated mouse retinal RNAs in this work, we investigated the expression features of MRLGs using the time-course transcriptomes. On the one hand, we revealed high temporal specificity of MRLGs expression. The lncRNAs extensively varied in quantity and content in developing retinas, such as the number and content of the detected MRLGs varied more than 50% between P1 and P14 retinas. In addition, the emphasis of MRLGs’ roles shifted from the protein synthesis/transport and autophagic pathways to visual perception associated processes along with retina development. On the other hand, we found the MRLG expression was orderly regulated during retina development. The expression patterns were more similar in developmental retinas at adjacent stages. According to the timeline, they were clustered into two main groups (i.e., embryonic group and neonatal group) and several subgroups (i.e., E12.5-E13.5, E14.5-E17.5, E18.5-P7, and P14-P28). These facts indicated that the MRLGs were undergone precise transcriptional regulation and their biological roles were continuously modulated during the neural retina formation.

Unexpectedly, MRLG expression at P1 was more similar to E12.5/E13.5 than P0/P3, possibly ascribed to the fact that many MRLGs were down-regulated at or before the P0 stage (e.g., E17.5 and E18.5) and up-regulated at P1. The genesis of retinal cell types ganglion (RGC), horizontal, cone, and amacrine mainly takes place before mouse birth, while the genesis of the rod, bipolar, and müller continues about two weeks after mouse birth. Thus, the modulation of lncRNA expression at P1 was possibly associated with the different stat of cell type birth. However, this can hardly explain that P1 retinas showed a similar MRLG expression pattern with E12.5/E13.5 retinas. For example, all six neuron types are produced starting from the early embryonic days, which means the differential expression of those MRLGs around birth is not, at least mainly, due to the birth of different cell types. We hypothesized that the extensive modulation of MRLG expression in P1 retinas resulted from response to light conditions, but obviously, more future efforts are required.

Moreover, we also revealed high spatial specificity of MRLG expression, e.g., according to the analyses of collected data, it seems about half MRLGs were specifically expressed in retinas. A comparative study of the lncRNA content between the retina and the other tissues also indicated the close association of MRLGs with the physiological roles of the retina tissue. For example, the cerebrum of the nervous system expressed more MRLGs than others, although only one library was sequenced for the cerebrum in contrast to up to ten for the others, indicating that the MRLGs only shared by the retina and cerebrum were very possibly involved in regulating neurogenesis.

### Potential roles of MRLGs in regulating differentiation of retinal neuron types

Based on the annotated MRLGs and public dataset, we investigated the lncRNA expression in mutants with abnormal retinal neurons that clues the involvement of several MRLGs in regulating retinal neurogenesis.

Math5-Brn3b/Isl1 pathway regulates the RGC specification and differentiation. Math5 is required in determining the RGC competence state. Its function loss leads to the lack of RGCs and optic nerves but would increase the abundance of cone photoreceptors [26–29]. In contrast, Brn3b and Isl1 function downstream of Math5 and co-regulate the RGC differentiation and survival by forming a complex [30, 31]. Previous work revealed that Math5, Brn3b and Isl1 regulated an overlapping but distinct group of downstream protein-coding genes [32]. On the contrary, we found that the differentially expressed MRLGs induced by the function loss of Brn3b, Isl1, and Math5 were almost non-overlapping. Similar results were also found when assessing the lncRNA expression in Oc1 or/and Oc2 mutants. It may indicate the high specificity of lncRNAs in transcription regulation or their cell type expression.

Exceptionally, we found *XLOC_013893*, the potential *cis*-regulator of *Irx3*, was down-regulated after function loss of Math5, Brn3b or Isl1. The exact function of Irx3 in retina development has not been determined. Nevertheless, derived from an early study, Irx3 in mouse and its homolog *Xiro3* in Xenopus are activated in the early embryo and expressed in neural progenitor cells [33]. Together with our findings herein, these facts indicated a role of *XLOC_013893* in RGC fate determination and survival through regulating the *cis*-target *Irx3*. In addition, *XLOC_010659* was down-regulated in both Math5- and Oc1-null mutants, which might be consistent with the role of Math5 and Oc1 in regulating the differentiation of early cell types, and also indicated that *XLOC_010659* was possibly involved in the early embryo development.

We also found some lncRNAs were differentially regulated in individual mutants. For instance, *XLOC_011005* was down-regulated in Oc1- and Math5-null mutants but was up-regulated in NRL-null mice, *XLOC_011568* was up-regulated in Oc1-null mutant but was down-regulated after function loss of Math5, and *XLOC_011622* was up-regulated in Oc1and Oc2 DKO but was down-regulated in NRL-null mutants. All these facts demonstrated that lncRNAs might be specifically involved in regulating the differentiation or development of retinal neuron cell types.

In summary, we identified dozens of differentially expressed intergenic MRLGs after the function loss of Math5, Brn3b, Isl1, Oc1, Oc2, and NRL. Although biological functions for almost all these MRLGs remain unclear, the *trans-* or *cis-*targets based function prediction and their differential expression stats suggested several valuable candidates for future exploration of the lncRNAs’ role in retinal neuron differentiation and development.

## Conclusions

This work generated a comprehensive annotation of mouse retinal lncRNAs, summarized their genomic and expression features, and revealed some of their potential functions and functions. We hope the presented work could promote our future understanding of retina neurogenesis and the regulatory roles of lncRNAs.

## Materials and methods

### Animals

All the animal experiments of this study were approved by the Ethics Committee for Experimental Animals of Hangzhou Normal University (permit number: 2018069). C57BL/6 mice were used in this study. The mice were euthanatized by carbon dioxide followed by cervical dislocation. The retinas were dissected from the embryonic (E12.5 to E18.5) and the neonatal mice (P0, P3, P5, P7, P14, P21, and P28) with the method described previously [19]. Retina tissues in each sample were isolated from at least three individuals. Two biological replicates were prepared for each developmental stage.

### Library construction and lncRNA-seq

Isolation and quality control of total RNAs was performed with the methods previously described (Wan et al., 2019). The strand-specific library was constructed mainly according to the protocols contributed by Zhong et al. [34]. Shortly, oligodT beads were used to capture polyA-RNAs, which were then eluted and fragmented by fragmentation buffer. The first-strand cDNA was synthesized by using random hexamers and the second-strand cDNA was generated using dNTPs (with the dTTP replaced by dUTP), DNA Polymerase I and RNase H. Double-strand cDNAs were purified using AMPure XP beads and the dUTP-containing second strand was digested using USER enzyme after end-repair, dA-tailing and adapter ligation. PCR amplification was performed subsequently, with the products cleaned up using AMPure XP beads and then used for short reads sequencing on HiSeq platforms (Illumina, CA, USA). Library construction and sequencing were conducted by NOVOGENE (Beijing, China).

### Identification of retinal lncRNAs

The mouse retinal lncRNAs were annotated via a hybrid strategy that merged the annotation from mouse genome projects, lncRNA-seq data analyses, and full-length transcripts sequencing (iso-seq). The dataset and detailed methods are described below.

(1) The lncRNAs from genome annotation (VM22-lncRNAs). The mouse genome annotation of the reference was retrieved from GENCODE (vM22) [4], from which 18,978 lncRNA transcripts were collected and stored in GTF format for further use.

(2) The lncRNAs from lncRNA-seq and data analysis (SR-lncRNAs). The short reads generated from lncRNA-seq were mapped to the reference genome (mm10) using TopHat2 (v2.1.0), and the transcripts were re-constructed with Cufflinks (v2.2.1) [35]. Next, TACO (v0.7.3) was used to combine the established transcripts [36]. Transcripts shorter than 200 nt or overlapping with coding genes’ exon regions were filtered out before further analyses. CNCI was then used to calculate the coding potential of the remaining transcripts [37].

(3) The lncRNAs from iso-seq and analysis (LR-lncRNAs). In our previous work, mixed RNAs of developing mouse retinas (E12.5 to P28) were sequenced with the iso-seq method, and 5,404 multiple-exon encoded lncRNAs were collected (Wan, 2019). In this work, we re-evaluated these candidates before further use. First, the short reads generated from lncRNA-seq were aligned to these transcripts with Bowtie (1.1.2) [38]. Next, the transcription direction was determined if more than two-fold short reads were aligned to one strand than the other (χ^2^-test, p-value ≤ 0.05). Then, we further checked the stranded transcripts and filtered out those shared one or more introns with protein-coding genes.

The lncRNA transcripts collected from genome annotation, lncRNA-seq and iso-seq were then merged with cuffmerge. Notably, lncRNAs that were lowly expressed (FPKM < 1) in all developmental stages were not included in further analyses. The detailed methods for calculating the gene expression level are described in the following sections. Finally, cuffcompare was used to evaluate the contribution of VM22-, SR-, and LR-lncRNAs to the final gene sets.

### Classification of retinal lncRNAs

Herein, we classify the annotated retinal lncRNAs into five groups based on their positional relationships with the flanking protein-coding genes. The antisense lncRNAs overlap the protein-coding gene on the opposite strand with more than one base. The intronic lncRNAs mainly arise from the introns of protein-coding genes. The divergent and convergent lncRNAs locate within 1 kb away from their nearest coding genes and are transcribed from the different and same strand, respectively. Finally, the strict intergenic lncRNAs refer to those distant from their nearest protein-coding genes (> 1 kb).

### Conservation analyses

The 60-way vertebrate alignment and conservation track was downloaded from the UCSC Genome Browser (http://genome.ucsc.edu/). Then, based on the conservation scores (phastcons), we assessed the conservation of exons from protein-coding genes and lncRNA genes, as well as random genomic regions (with an arbitrary length of less than 1 kb and the same number as the exons of lncRNA).

BLASTN was used to search the homologs of mouse retinal lncRNAs in several vertebrates [39], i.e., zebrafish, rat, platypus, pig, orangutan, opossum, gorilla, cow, chimp, chicken and human, for which the lncRNAs were retrieved from NONCODE database (v5) [40].

### Spatiotemporal expression analyses

To analyze the protein-coding gene and lncRNA gene expression in developing retinas, we combined the information on protein-coding genes from mouse genome annotation (VM22) and the lncRNAs from this work in a new GTF file. TopHat2 (v2.1.0) and Cufflinks (v2.2.1) were used for short reads mapping and expression level calculation. The expression levels of the lncRNA genes were merged in an FPKM matrix. Before further analyses, the lowly expressed genes (FPKM < 1 in all biological replicates) were removed. Then, the FPKM matrix was used for hierarchical clustering and principal component analyses (PCA) with the R tools stats and hclust (R version 3.6.3). The temporal expression specificity of MRLGs was estimated by the tau values with in-house developed Perl scripts [41].

RNA-seq data for another 15 mouse tissues were retrieved from Sequence Read Archive (SRA) (https://www.ncbi.nlm.nih.gov/sra), including those from the digestive system (liver, stomach, large intestine, small intestine, and colon), urinary system (kidney), endocrine system (thymus and adrenal), reproductive system (mammary gland, testis, and ovary), nervous system (cerebrum), respiratory system (lung), and circulation system (heart and spleen). The accession numbers of these datasets were collected and provided by Zhao and colleagues [42]. Expression levels of lncRNA genes in these tissues were calculated using the same strategy as mentioned above.

### Identification of *cis*- and *trans*-regulations

Gene expression correlation (Pearson correlation coefficient) between protein-coding and lncRNA genes was calculated using R tools, and the co-expression modules were established with WGCNA [43]. The *trans*-regulation was determined if the protein-coding gene and lncRNA gene showed strong expression correlation (*cor* ≥ 0.90) and were clustered into the same WGCNA module. Likewise, the *cis*-regulation between lncRNA and its target was determined if they were less than 100 kb distant and correlated in expression.

### Differential expression of lncRNA genes in retinas of gene knockout mice

To identify the differentially expressed genes (DEGs) after function loss of the transcription factors, we downloaded the corresponding RNA-seq data from SRA, including SRR1166785 and SRR1167631-SRR1167641 for Brn3b-null, Isl1-null, and Math5-null mutants (E14.5) [31], SRR1618614-SRR1618625 for Oc1-null, Oc2-null and Oc1/Oc2 double knockout mutants (E14.5) [22], as well as SRR358714-SRR358719 for NRL-null mutants (P21) [44]. Please consult their related literature to get more details about the experimental design and the data generation. After short reads alignment, the counts of mapped reads were estimated using HTSeq [45], and the DEGs were identified using DESeq2 (|log2foldchange| ≥ 1 and p-value ≤ 0.05 ) [46].

### Statistics analyses

The R language was used to perform the *chi*-square test and *t*-test in this work to estimate the significance of functional enrichment or the difference between groups.

## Electronic supplementary material

Below is the link to the electronic supplementary material.

Supplementary Material 1

Supplementary Material 2

Supplementary Material 3

Supplementary Material 4

Supplementary Material 5

Supplementary Material 6

Supplementary Material 7

Supplementary Material 8

Supplementary Material 9

Supplementary Material 10

Supplementary Material 11

Supplementary Material 12

Supplementary Material 13

Supplementary Material 14

Supplementary Material 15

## Data Availability

The datasets generated in this work have been deposited in National Genomics Data Center of China under the project PRJCA013588 (CRR622060-CRR622089).

## List of Abbreviations

DEG: Differentially expressed gene
DKO: Double knockout
lncRNA: Long non-coding RNAs
MRLG: Mouse retinal lncRNA gene
Oc1/2: Onecut1/2
RGC: Retinal ganglion cell
RNA-seq: RNA sequencing

## References

[1] Fatica A, Bozzoni I (2014). Long non-coding RNAs: new players in cell differentiation and development. Nat Rev Genet.

[2] Statello L, Guo CJ, Chen LL, Huarte M (2021). Gene regulation by long non-coding RNAs and its biological functions. Nat Rev Mol Cell Biol.

[3] Xu X, Liu S, Yang Z, Zhao X, Deng Y, Zhang G, Pang J, Zhao C, Zhang W (2021). A systematic review of computational methods for predicting long noncoding RNAs. Brief Funct Genomics.

[4] Frankish A, Diekhans M, Ferreira AM, Johnson R, Jungreis I, Loveland J, Mudge JM, Sisu C, Wright J, Armstrong J (2019). GENCODE reference annotation for the human and mouse genomes. Nucleic Acids Res.

[5] Zhao L, Wang J, Li Y, Song T, Wu Y, Fang S, Bu D, Li H, Sun L, Pei D (2021). NONCODEV6: an updated database dedicated to long non-coding RNA annotation in both animals and plants. Nucleic Acids Res.

[6] Gloss BS, Dinger ME (2016). The specificity of long noncoding RNA expression. Biochim Biophys Acta.

[7] Masland RH (2012). The neuronal organization of the retina. Neuron.

[8] Xiang M (2013). Intrinsic control of mammalian retinogenesis. Cell Mol Life Sci.

[9] Heavner W, Pevny L. Eye development and retinogenesis. Cold Spring Harb Perspect Biol 2012, 4(12). 10.1101/cshperspect.a008391 PMC3504437 23071378

[10] Rapicavoli NA, Poth EM, Zhu H, Blackshaw S (2011). The long noncoding RNA Six3OS acts in trans to regulate retinal development by modulating Six3 activity. Neural Dev.

[11] Rapicavoli NA, Poth EM, Blackshaw S (2010). The long noncoding RNA RNCR2 directs mouse retinal cell specification. BMC Dev Biol.

[12] Tan JY, Vance KW, Varela MA, Sirey T, Watson LM, Curtis HJ, Marinello M, Alves S, Steinkraus B, Cooper S (2014). Cross-talking noncoding RNAs contribute to cell-specific neurodegeneration in SCA7. Nat Struct Mol Biol.

[13] Yao J, Wang XQ, Li YJ, Shan K, Yang H, Wang YN, Yao MD, Liu C, Li XM, Shen Y (2016). Long non-coding RNA MALAT1 regulates retinal neurodegeneration through CREB signaling. EMBO Mol Med.

[14] Chen X, Jiang C, Qin B, Liu G, Ji J, Sun X, Xu M, Ding S, Zhu M, Huang G (2017). LncRNA ZNF503-AS1 promotes RPE differentiation by downregulating ZNF503 expression. Cell Death Dis.

[15] Wan P, Su W, Zhuo Y (2017). Precise long non-coding RNA modulation in visual maintenance and impairment. J Med Genet.

[16] Sun LF, Chen XJ, Jin ZB (2020). Emerging roles of non-coding RNAs in retinal diseases: a review. Clin Exp Ophthalmol.

[17] Chen W, Yang S, Zhou Z, Zhao X, Zhong J, Reinach PS, Yan D (2017). The Long Noncoding RNA Landscape of the Mouse Eye. Investig Ophthalmol Vis Sci.

[18] Chen G, Qian HM, Chen J, Wang J, Guan JT, Chi ZL (2021). Whole transcriptome sequencing identifies key circRNAs, lncRNAs, and miRNAs regulating neurogenesis in developing mouse retina. BMC Genomics.

[19] Wan Y, Liu X, Zheng D, Wang Y, Chen H, Zhao X, Liang G, Yu D, Gan L (2019). Systematic identification of intergenic long-noncoding RNAs in mouse retinas using full-length isoform sequencing. BMC Genomics.

[20] Stumpo DJ, Bock CB, Tuttle JS, Blackshear PJ (1995). MARCKS deficiency in mice leads to abnormal brain development and perinatal death. Proc Natl Acad Sci USA.

[21] Shibasaki K, Takebayashi H, Ikenaka K, Feng L, Gan L (2007). Expression of the basic helix-loop-factor Olig2 in the developing retina: Olig2 as a new marker for retinal progenitors and late-born cells. Gene Expr Patterns.

[22] Sapkota D, Chintala H, Wu F, Fliesler SJ, Hu Z, Mu X (2014). Onecut1 and Onecut2 redundantly regulate early retinal cell fates during development. Proc Natl Acad Sci USA.

[23] Levin JZ, Yassour M, Adiconis X, Nusbaum C, Thompson DA, Friedman N, Gnirke A, Regev A (2010). Comprehensive comparative analysis of strand-specific RNA sequencing methods. Nat Methods.

[24] Krappinger JC, Bonstingl L, Pansy K, Sallinger K, Wreglesworth NI, Grinninger L, Deutsch A, El-Heliebi A, Kroneis T, McFarlane RJ (2021). Non-coding natural antisense transcripts: analysis and application. J Biotechnol.

[25] Ayupe AC, Beckedorff F, Levay K, Yon B, Salgueiro Y, Shiekhattar R, Park KK (2021). Identification of long noncoding RNAs in injury-resilient and injury-susceptible mouse retinal ganglion cells. BMC Genomics.

[26] Yang Z, Ding K, Pan L, Deng M, Gan L (2003). Math5 determines the competence state of retinal ganglion cell progenitors. Dev Biol.

[27] Brown NL, Patel S, Brzezinski J, Glaser T (2001). Math5 is required for retinal ganglion cell and optic nerve formation. Development.

[28] Le TT, Wroblewski E, Patel S, Riesenberg AN, Brown NL (2006). Math5 is required for both early retinal neuron differentiation and cell cycle progression. Dev Biol.

[29] Feng L, Xie ZH, Ding Q, Xie X, Libby RT, Gan L (2010). MATH5 controls the acquisition of multiple retinal cell fates. Mol Brain.

[30] Pan L, Deng M, Xie X, Gan L (2008). ISL1 and BRN3B co-regulate the differentiation of murine retinal ganglion cells. Development.

[31] Li R, Wu F, Ruonala R, Sapkota D, Hu Z, Mu X (2014). Isl1 and Pou4f2 form a complex to regulate target genes in developing retinal ganglion cells. PLoS ONE.

[32] Mu X, Fu X, Beremand PD, Thomas TL, Klein WH (2008). Gene regulation logic in retinal ganglion cell development: Isl1 defines a critical branch distinct from but overlapping with Pou4f2. Proc Natl Acad Sci USA.

[33] Bellefroid EJ, Kobbe A, Gruss P, Pieler T, Gurdon JB, Papalopulu N (1998). Xiro3 encodes a Xenopus homolog of the Drosophila Iroquois genes and functions in neural specification. EMBO J.

[34] Zhong S, Joung JG, Zheng Y, Chen YR, Liu B, Shao Y, Xiang JZ, Fei Z, Giovannoni JJ (2011). High-throughput illumina strand-specific RNA sequencing library preparation. Cold Spring Harbor Protoc.

[35] Trapnell C, Roberts A, Goff L, Pertea G, Kim D, Kelley DR, Pimentel H, Salzberg SL, Rinn JL, Pachter L (2012). Differential gene and transcript expression analysis of RNA-seq experiments with TopHat and Cufflinks. Nat Protoc.

[36] Niknafs YS, Pandian B, Iyer HK, Chinnaiyan AM, Iyer MK (2017). TACO produces robust multisample transcriptome assemblies from RNA-seq. Nat Methods.

[37] Sun L, Luo H, Bu D, Zhao G, Yu K, Zhang C, Liu Y, Chen R, Zhao Y (2013). Utilizing sequence intrinsic composition to classify protein-coding and long non-coding transcripts. Nucleic Acids Res.

[38] Langmead B, Salzberg SL (2012). Fast gapped-read alignment with Bowtie 2. Nat Methods.

[39] Altschul SF, Gish W, Miller W, Myers EW, Lipman DJ (1990). Basic local alignment search tool. J Mol Biol.

[40] Fang S, Zhang L, Guo J, Niu Y, Wu Y, Li H, Zhao L, Li X, Teng X, Sun X (2018). NONCODEV5: a comprehensive annotation database for long non-coding RNAs. Nucleic Acids Res.

[41] Yanai I, Benjamin H, Shmoish M, Chalifa-Caspi V, Shklar M, Ophir R, Bar-Even A, Horn-Saban S, Safran M, Domany E (2005). Genome-wide midrange transcription profiles reveal expression level relationships in human tissue specification. Bioinformatics.

[42] Zhao Y, Liu W, Zeng J, Liu S, Tan X, Aljohi H, Hu S (2016). Identification and analysis of mouse non-coding RNA using transcriptome data. Sci China Life Sci.

[43] Langfelder P, Horvath S (2008). WGCNA: an R package for weighted correlation network analysis. BMC Bioinformatics.

[44] Brooks MJ, Rajasimha HK, Roger JE, Swaroop A (2011). Next-generation sequencing facilitates quantitative analysis of wild-type and Nrl(-/-) retinal transcriptomes. Mol Vis.

[45] Anders S, Pyl PT, Huber W (2015). HTSeq–a Python framework to work with high-throughput sequencing data. Bioinformatics.

[46] Love MI, Huber W, Anders S (2014). Moderated estimation of fold change and dispersion for RNA-seq data with DESeq2. Genome Biol.

