# Supplementary figures and images for: The landscape of the long non-coding RNAs in developing mouse retinas
Source: BMC Genomics. 2023 May 10;24:252. doi: 10.1186/s12864-023-09354-w (PMC10173636; doi:10.1186/s12864-023-09354-w)

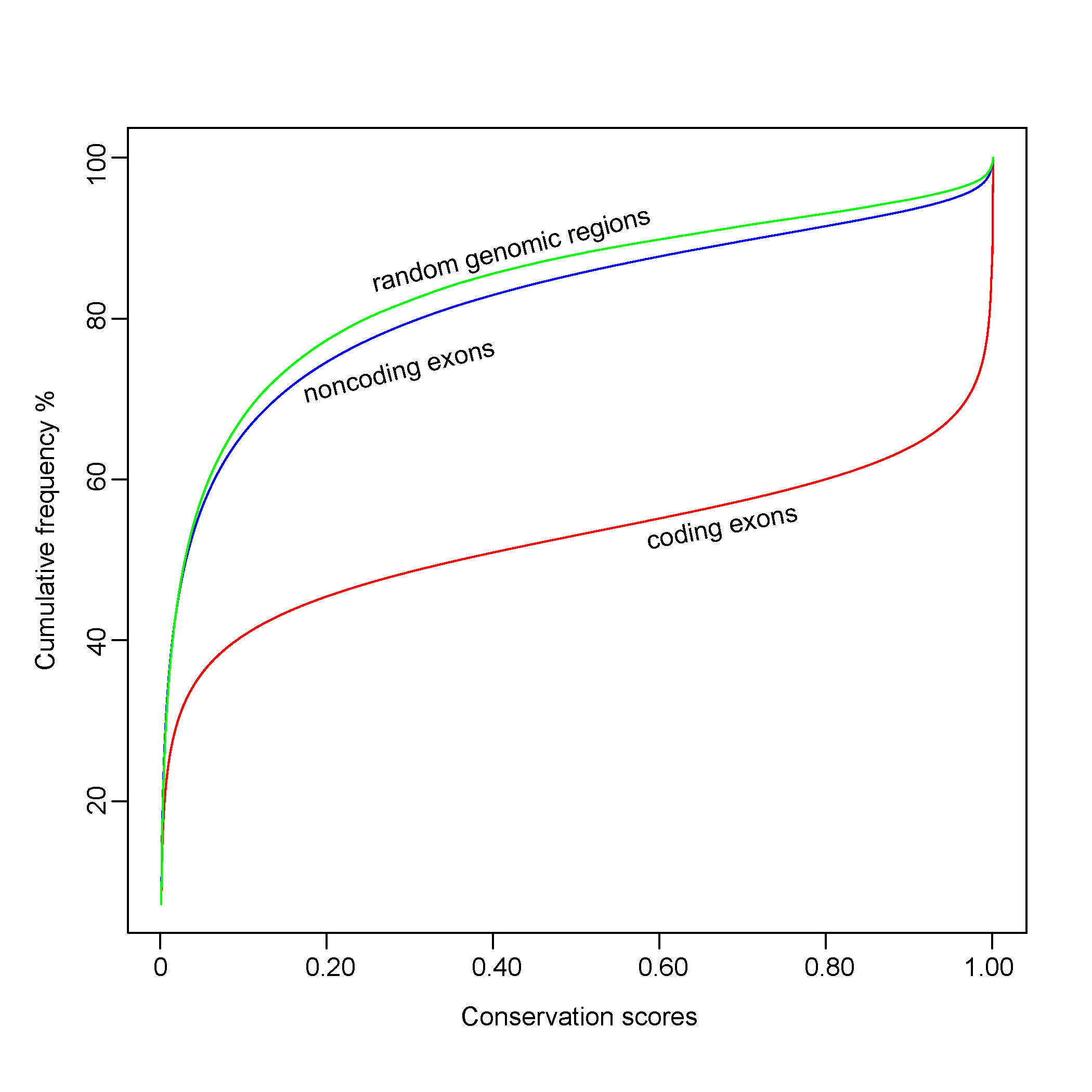

Supplement: Supplementary file 1 — Supplementary Material 1 [file 12864_2023_9354_MOESM1_ESM.tif]

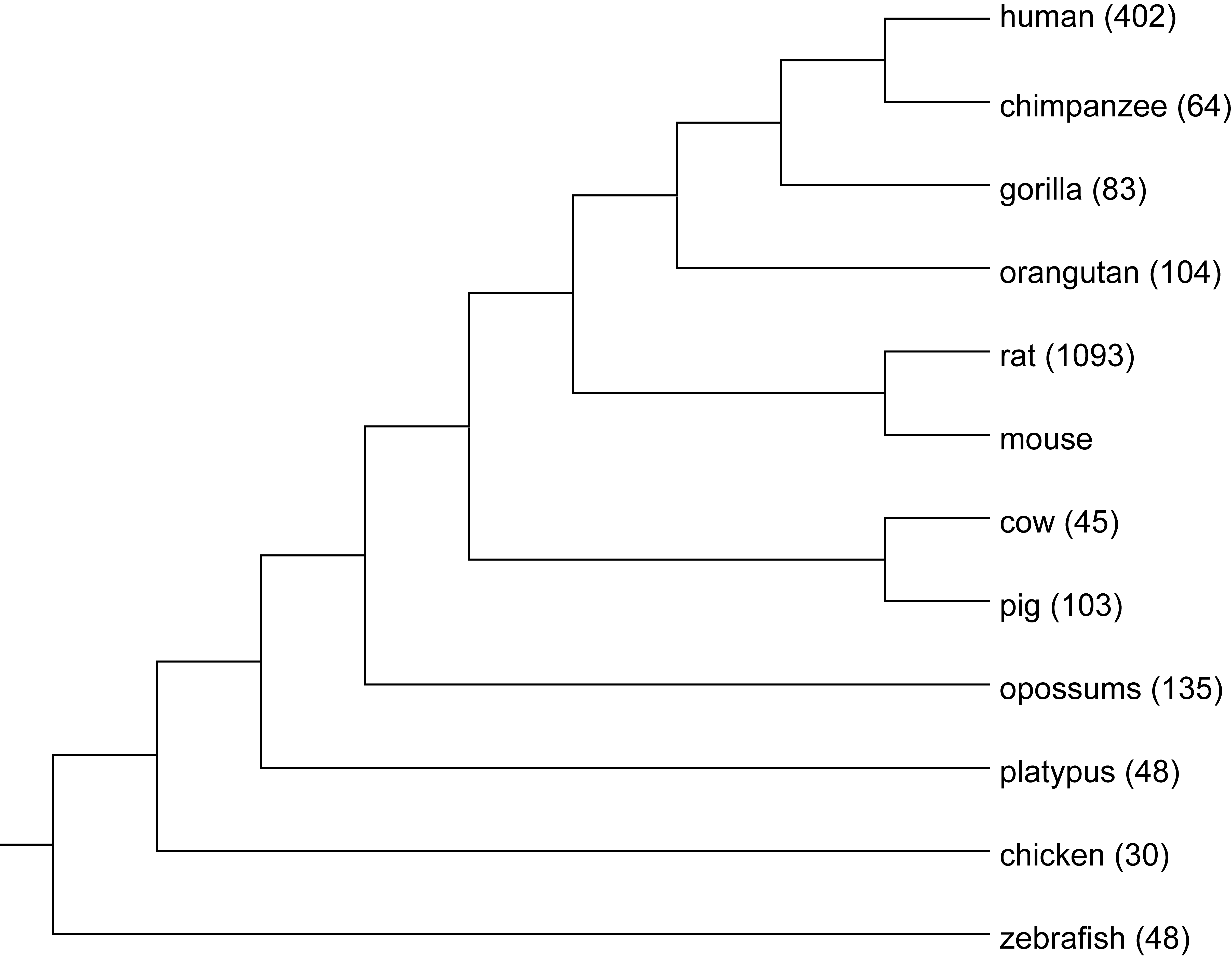

Supplement: Supplementary file 2 — Supplementary Material 2 [file 12864_2023_9354_MOESM2_ESM.tif]

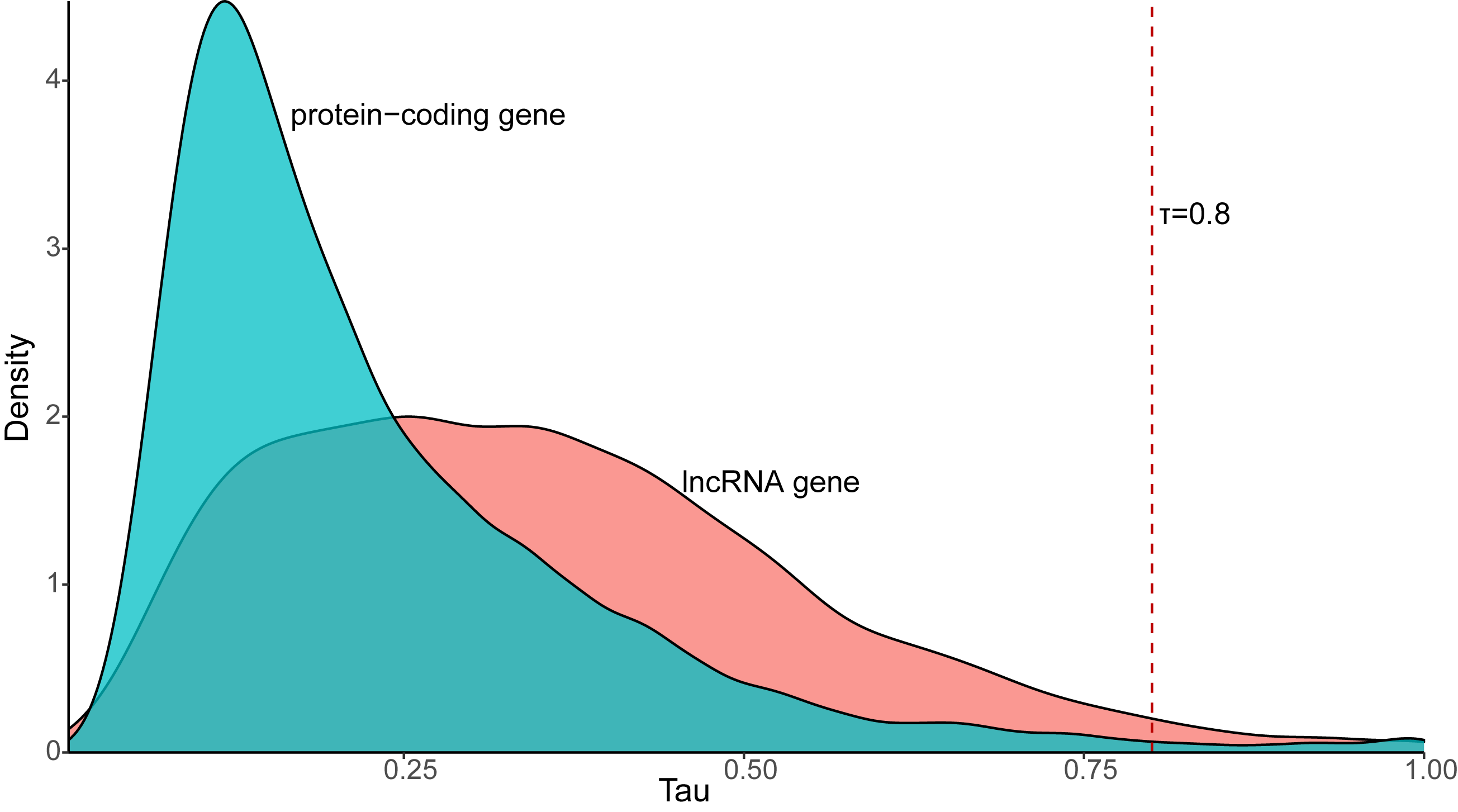

Supplement: Supplementary file 3 — Supplementary Material 3 [file 12864_2023_9354_MOESM3_ESM.tif]

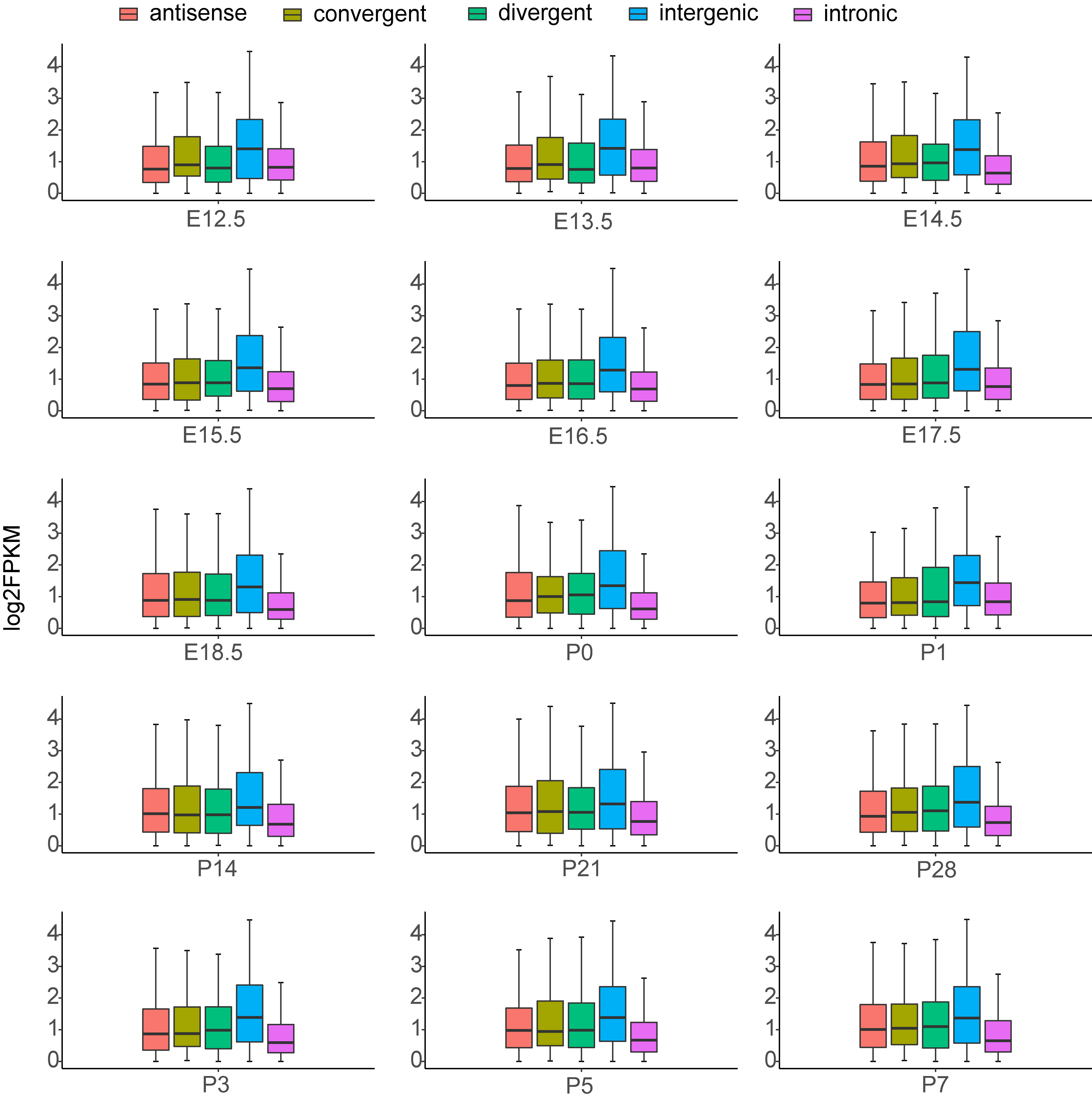

Supplement: Supplementary file 4 — Supplementary Material 4 [file 12864_2023_9354_MOESM4_ESM.tif]

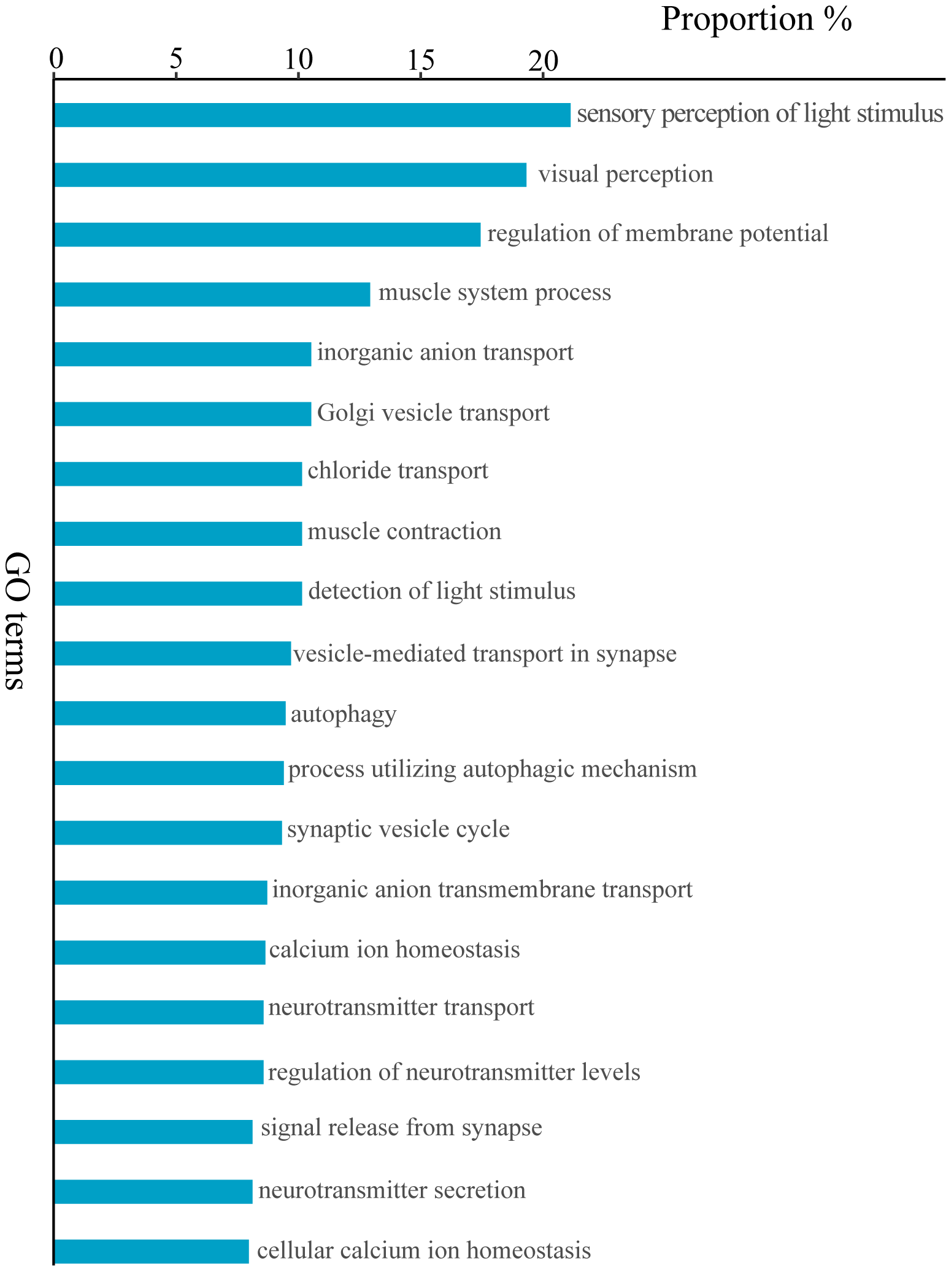

Supplement: Supplementary file 5 — Supplementary Material 5 [file 12864_2023_9354_MOESM5_ESM.tif]

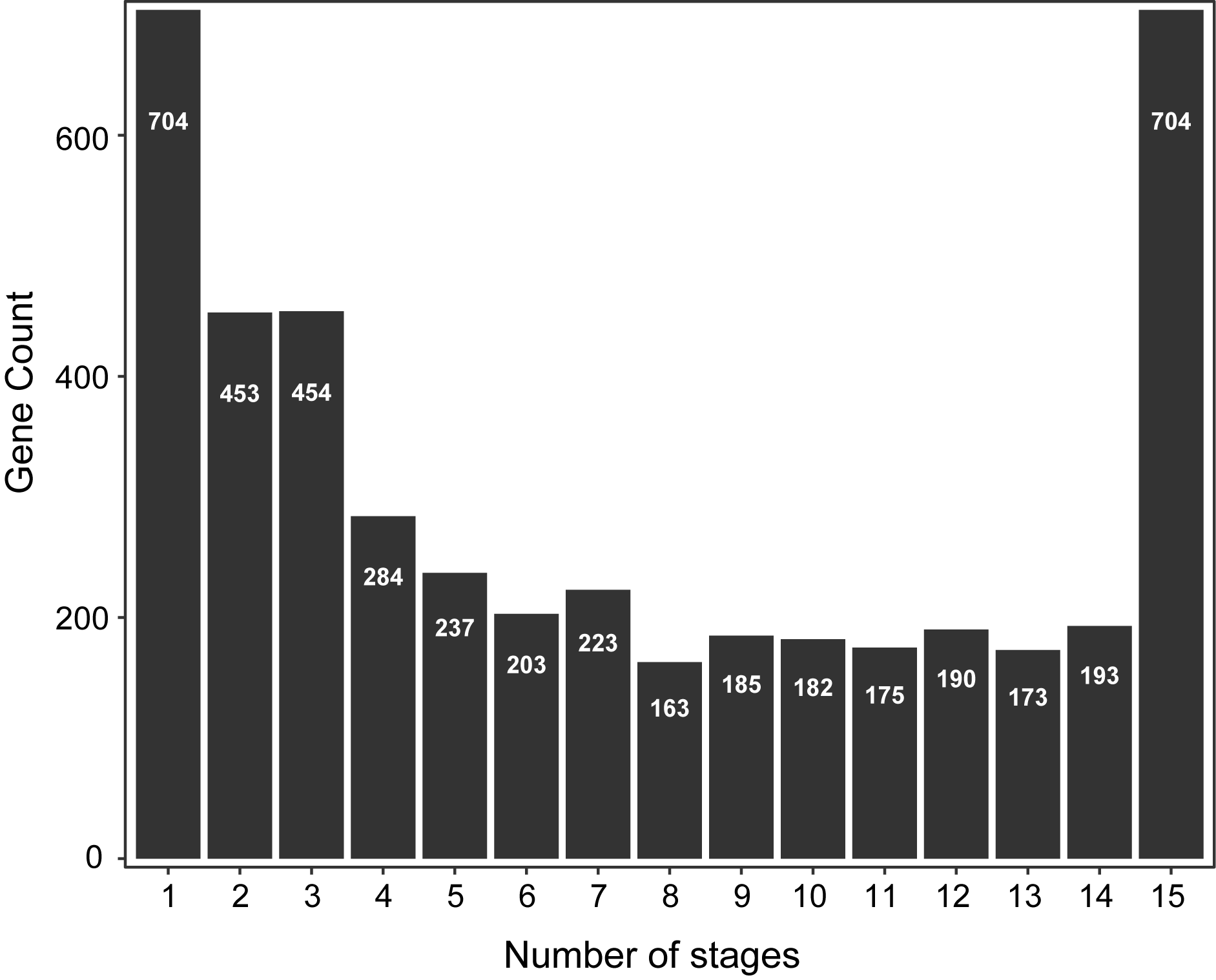

Supplement: Supplementary file 6 — Supplementary Material 6 [file 12864_2023_9354_MOESM6_ESM.tif]

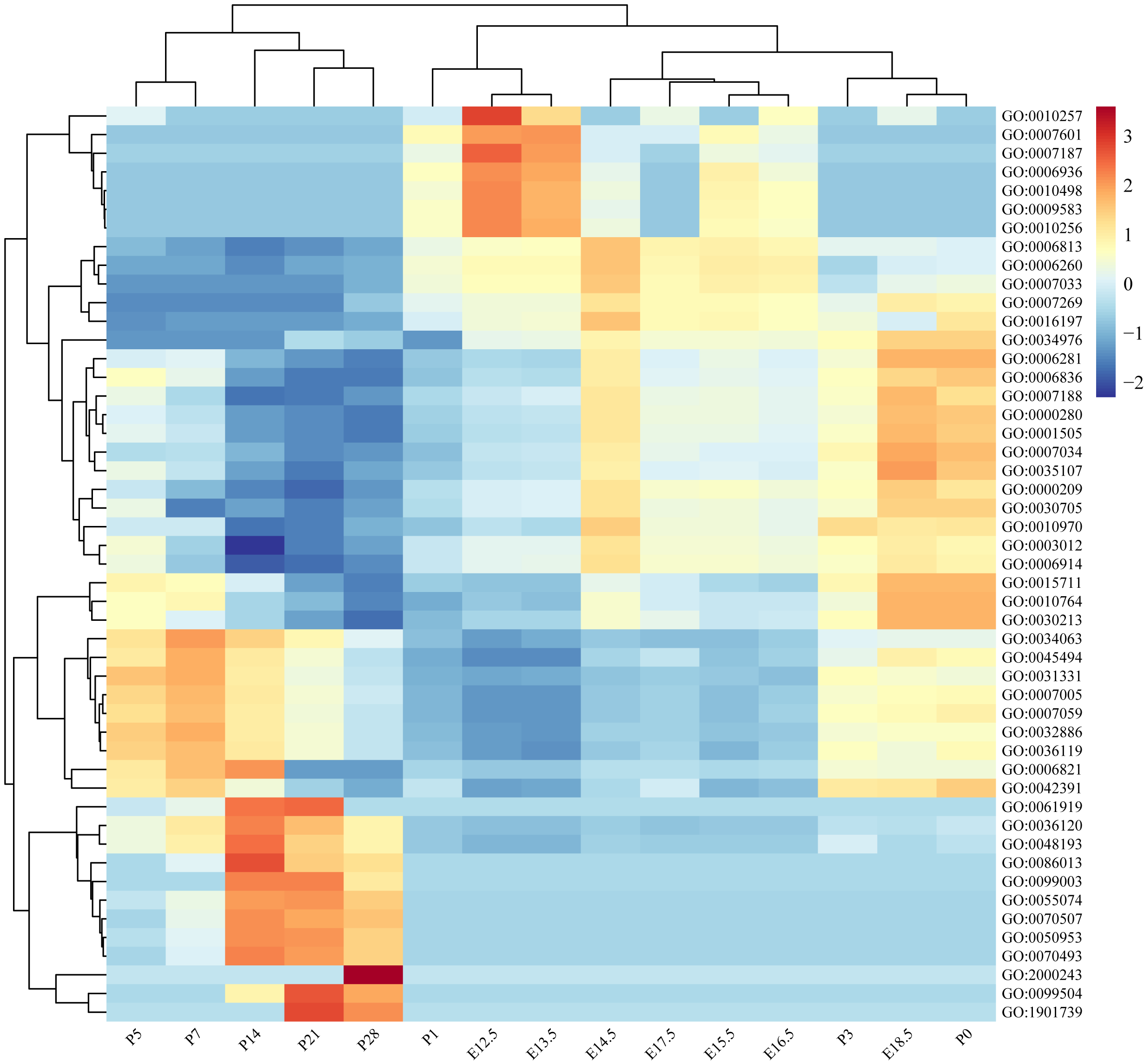

Supplement: Supplementary file 7 — Supplementary Material 7 [file 12864_2023_9354_MOESM7_ESM.tif]

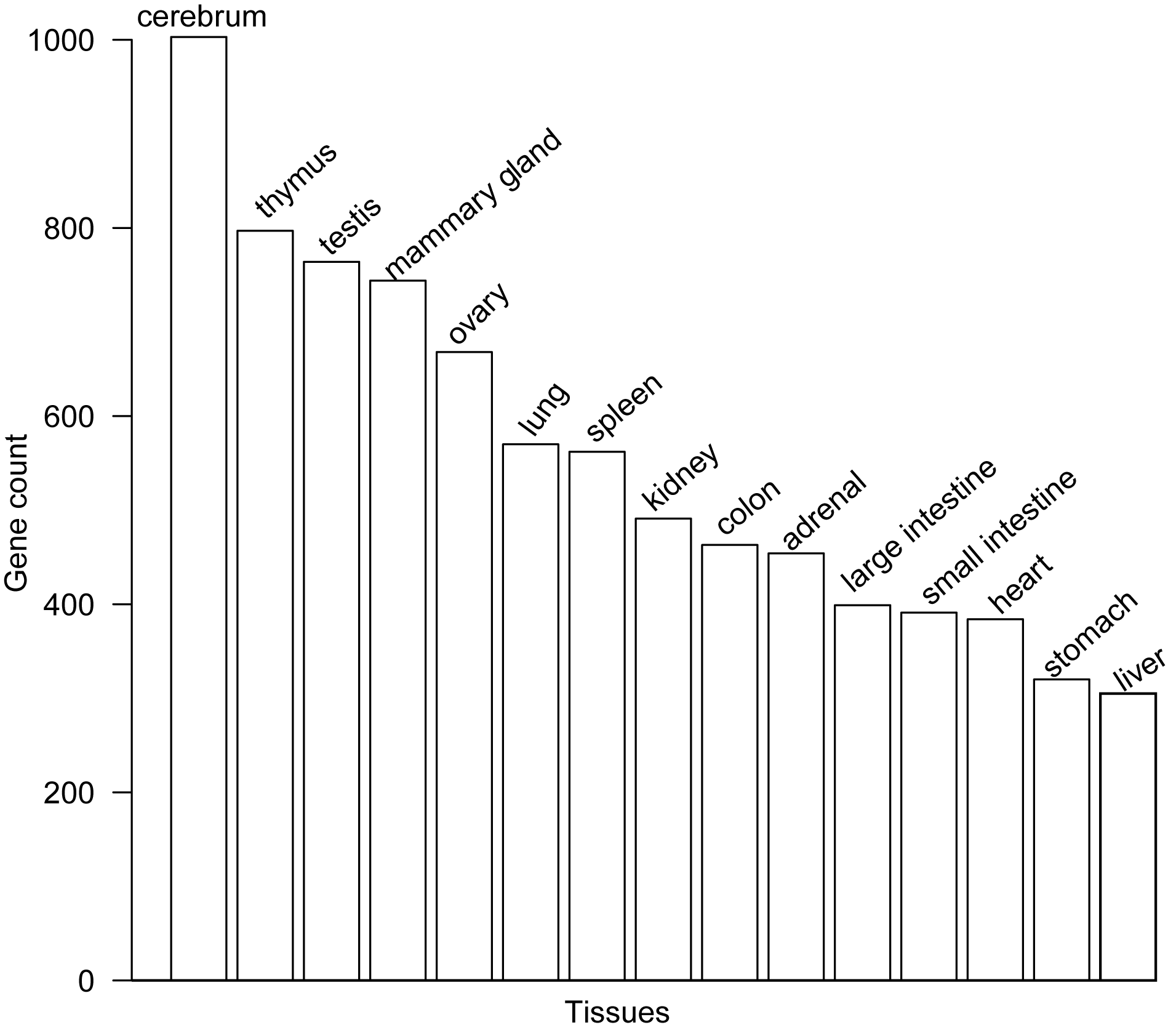

Supplement: Supplementary file 8 — Supplementary Material 8 [file 12864_2023_9354_MOESM8_ESM.tif]

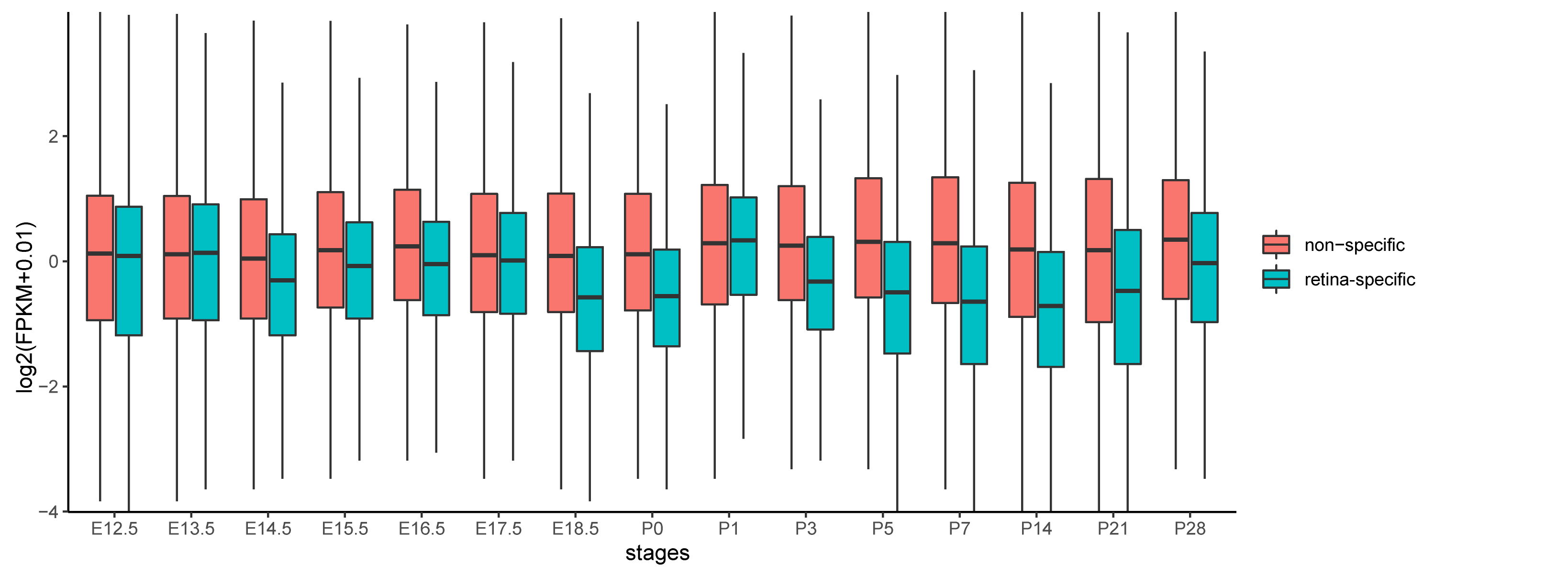

Supplement: Supplementary file 9 — Supplementary Material 9 [file 12864_2023_9354_MOESM9_ESM.tif]
