# Supplementary material for: The landscape of the long non-coding RNAs in developing mouse retinas
Source: BMC Genomics. 2023 May 10;24:252. doi: 10.1186/s12864-023-09354-w (PMC10173636; doi:10.1186/s12864-023-09354-w)
Supplement: Supplementary file 15 — Supplementary Material 15 [file 12864_2023_9354_MOESM15_ESM.docx]

## Supplementary Materials

**Figure S1**. Genomic conservation of lncRNA exons. Conservation of exons of protein-coding genes and lncRNA genes, as well as random genomic regions were compared based on the conservation scores (phastcons).

**Figure S2**. The lncRNAs conserved across vertebrates. A primary sequence-based homology search was performed to identify the conserved lncRNAs across vertebrates (≥ identity 85% within ≥ 100 bp regions). The number after species names indicated the number of conserved lncRNAs.

**Figure S3**. The temporal specificity of lncRNA expression in developing retinas. The Tau value of 0.8 was used to specify the specifically expressed genes.

**Figure S4**. Comparison of the expression of different classes of lncRNAs. According to the relative position with the nearest protein-coding genes, lncRNAs were classified as antisense (overlap), intronic (within intron regions), divergent/convergent (within 1 kb up- or down-stream) and intergenic lncRNAs (> 1 kb distant).

**Figure S5**. Top twenty abundant GO terms for trans-acting mouse retinal lncRNAs.

**Figure S6**. Expressed mouse retinal lncRNAs in developing retinas. The lncRNA genes were counted in particular stage if they were expressed at a level of FPKM ≥ 1.

**Figure S7**. Clustering analyses of the functions of expressed *trans*-acting lncRNAs in developing retinas.

**Figure S8**. Statistics of the retinal lncRNAs that expressed in the other 15 tissues. The lncRNAs were considered expressed if they were expressed at a level of FPKM ≥ 1 in at least one library.

**Figure S9**. Expression of lncRNAs specific and non-specific to retina tissue during retina development.

**Table S1**. The lncRNA genes expressed in developing mouse retinas (E12.5-P28). Only the lncRNA genes expressed at a level of FPKM ≥ 1 in at least one developmental stage were included.

**Table S2**. The *cis*-regulations between lncRNA and protein-coding genes in developing mouse retinas. The cis-target locates within 100 kb up-/down-stream of the regulatory lncRNA gene and are correlated in expression (*cor* ≥ 0.9).

**Table S3**. Pairwise comparison of expressed lncRNAs between different stages. The average FPKM values of biological replicates were used to classify the lncRNAs as expressed (FPKM ≥ 1) or not in individual stages.

**Table S4**. The biological functions of *trans*-acting lncRNAs in developing retinas. GO terms that represent more than 5% of expressed *trans*-acting lncRNAs were included in the table.

**Table S5**. The differentially expressed lincRNAs after function loss of transcription factors involved in regulating retinal neurogenesis. The sign ‘—’ in column ‘Symbol’ indicated novel lncRNAs that were identified in this work, and ‘/’ indicated the annotation of related lncRNAs overlapped more than one loci in genome annotation. DKO: double knockout.
